# Supplementary material for: Soft matrix promotes immunosuppression in tumor-resident immune cells via COX-FGF2 signaling
Source: Nat Commun. 2025 May 27;16:4908. doi: 10.1038/s41467-025-60092-x (PMC12116891; doi:10.1038/s41467-025-60092-x)
Supplement: Supplementary file 1 — Supplementary Information [file 41467_2025_60092_MOESM1_ESM.pdf]

# Supplementary Information

# Soft Matrix Promotes Immunosuppression in Tumor-Resident Immune Cells via COX-FGF2 Signaling

## Supplementary Figure 1.

A

| Gel | Storage modulus G´ (Pa) | Loss modulus G´´ (Pa) | Phase angle δ (degrees) |
|---|---|---|---|
| Stiff NC | 978 ± 333 | 142 ± 47 | 8.3 ± 0.3 |
| Soft NC | 12 ± 2.6 | 2.4 ± 0.6 | 11.3 ±1.5 |
| Stiff PG | 6470 ± 1661 | 878 ± 183 | 7.8 ± 0.4 |
| Soft PG | 150 ± 12.1 | 50 ± 2.8 | 19 ± 1.3 |

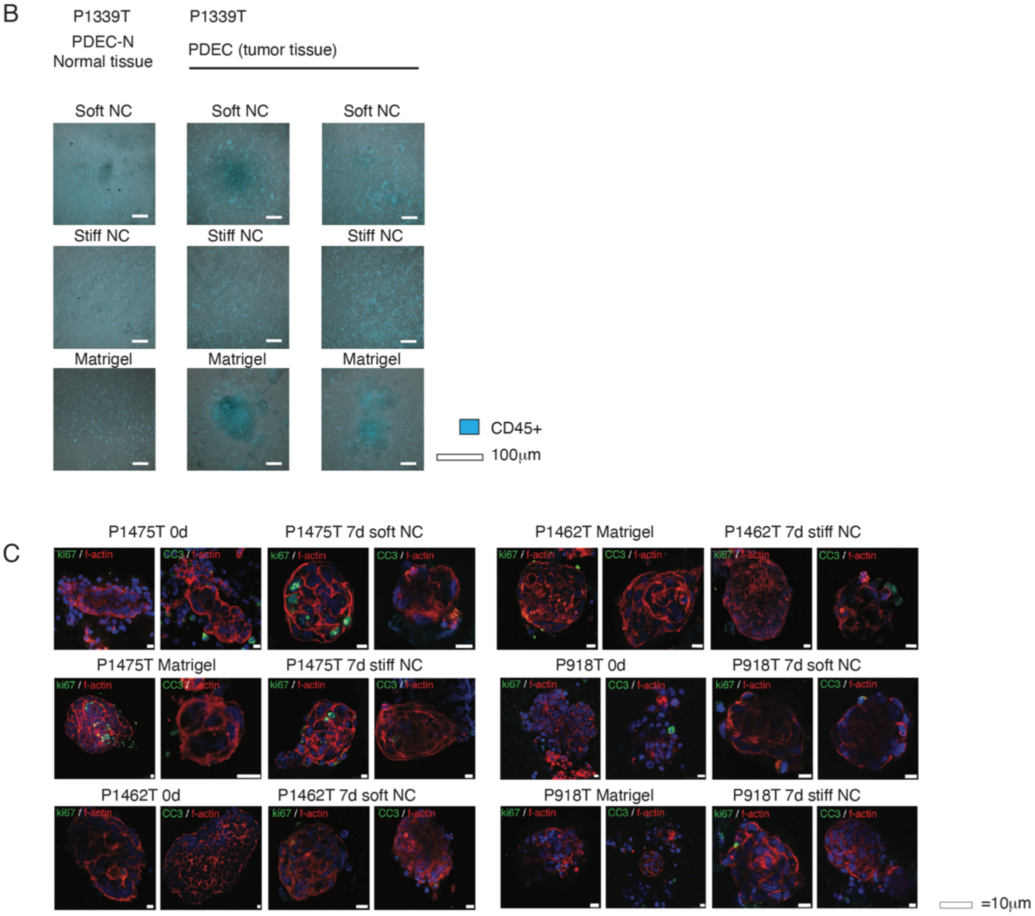


**Supplementary Figure 1. a,** Viscoelastic properties of stiff and soft nanocellulose (NC) and PeptiGel (PG) gels measured with oscillatory rheometer. (NC n=5, PG n=3). **b,** Live immune cell staining with fluorescent CD45 antibody (blue) in the soft and stiff NC gels or in Matrigel cultured patient derived explant cultures (PDEC) after 6 days of *ex vivo* culture (P1339N/T). **c,** Immunofluorescent staining of cleaved caspase 3 (CC3), F-actin, and Ki67 from 3 individual patients. Samples were cultured in Matrigel as well as in soft and stiff NC gels for 7 days.

**Supplementary Figure 2.**

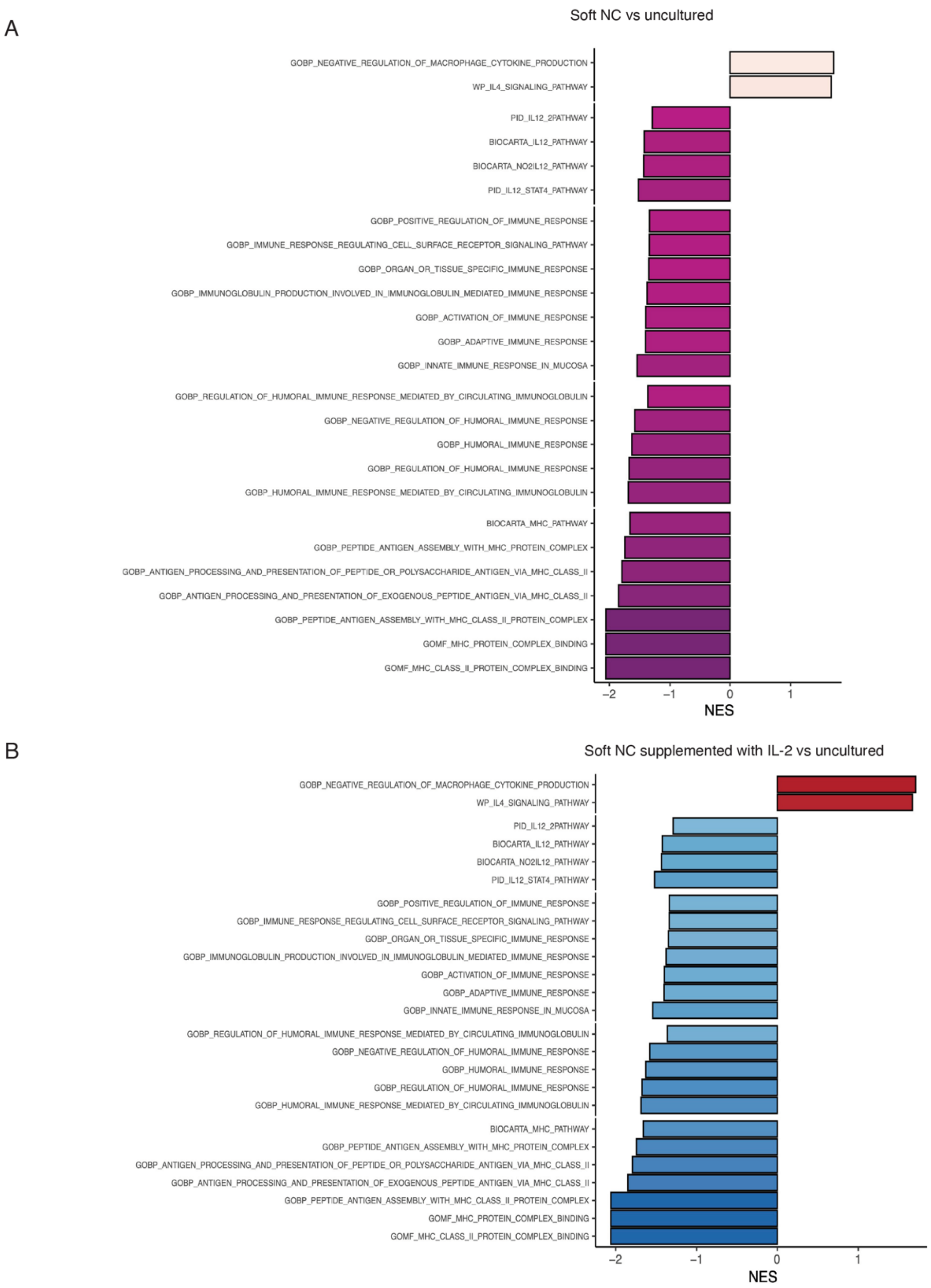


**Supplementary Figure 2. Gene set enrichment analysis. a,** Names for the gene sets in Figure 1 I. GSEA analysis of the differentially expressed gene sets in the soft nanocellulose (NC) gel (n=4) compared to the uncultured sample (n=5). **b,** Gene set enrichment analysis **(**GSEA) of the top

differentially expressed immunological gene sets between the uncultured sample and the IL-2 functionalized NC gel (GrowDex A supplemented with IL-2). FDR <0.2 (n=5).

**Supplementary Figure 3.**

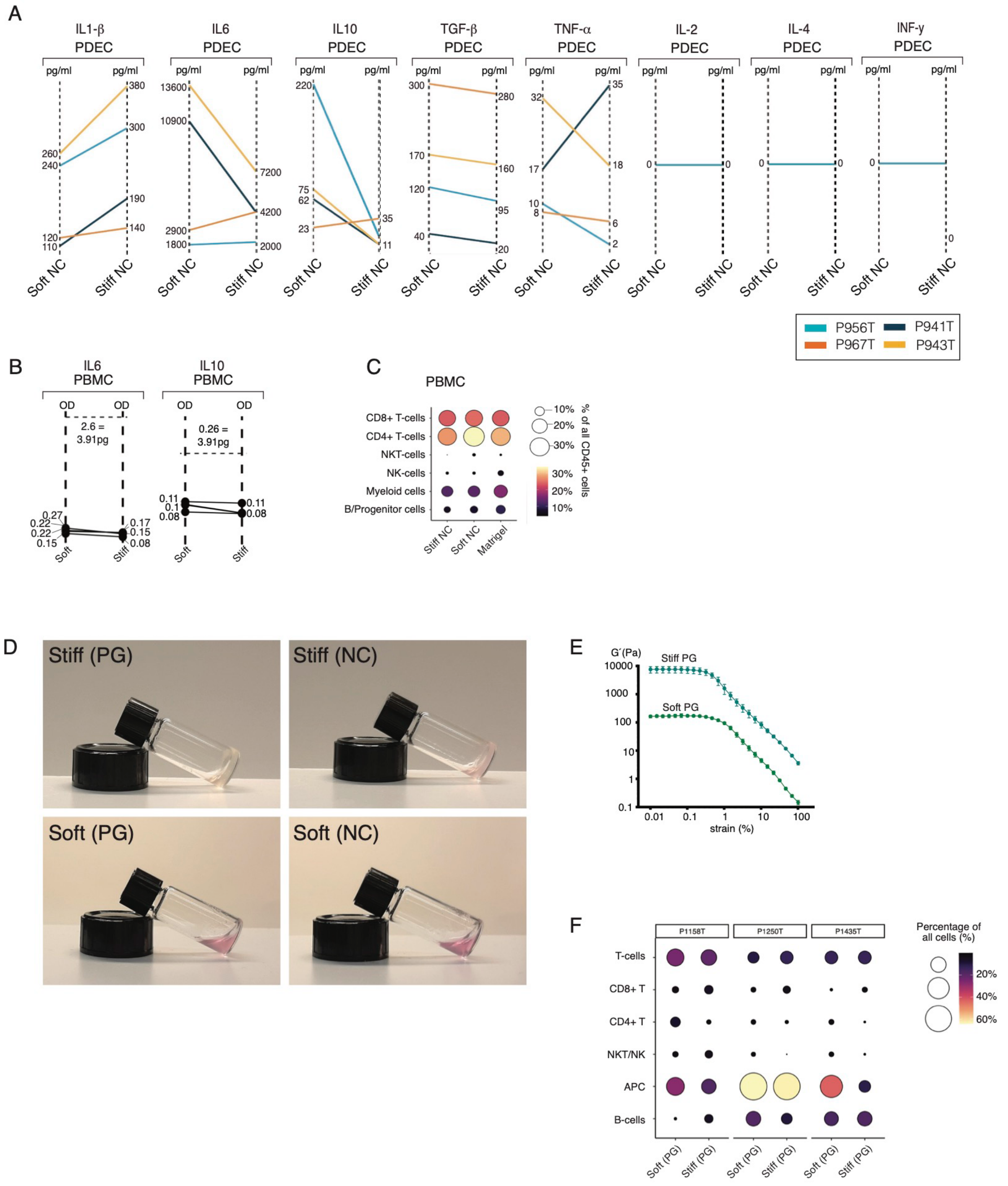

**Supplementary Figure 3. a,** ELISA cytokine analysis after 3 days of *ex vivo* patient derived explant cultures (PDEC) from four individual patients. **b,** ELISA cytokine analysis after 3 days of *ex vivo* peripheral blood mononuclear cell (PBMC) culture from four individual donors in the soft and stiff NC. Values fall below the standard curve and are hence represented as OD-values. **c,** Flow cytometry analysis of main cell types in PBMCs cultured in soft and stiff nanocellulose (NC) gels and in Matrigel for 3 days. Dot size and color represent the relative cell number from all cells (%). **d,** Vial inversion images of matrices used in the study. **e,** Strain-sweep measurement of used PeptiGel concentrations (n=3). Dots represent the medium and error bars represent the standard deviation. **f,** Main cell types present in the PeptiGel matrices after 3 days of culture, analyzed with flow cytometry.

**Supplementary Figure 4.**

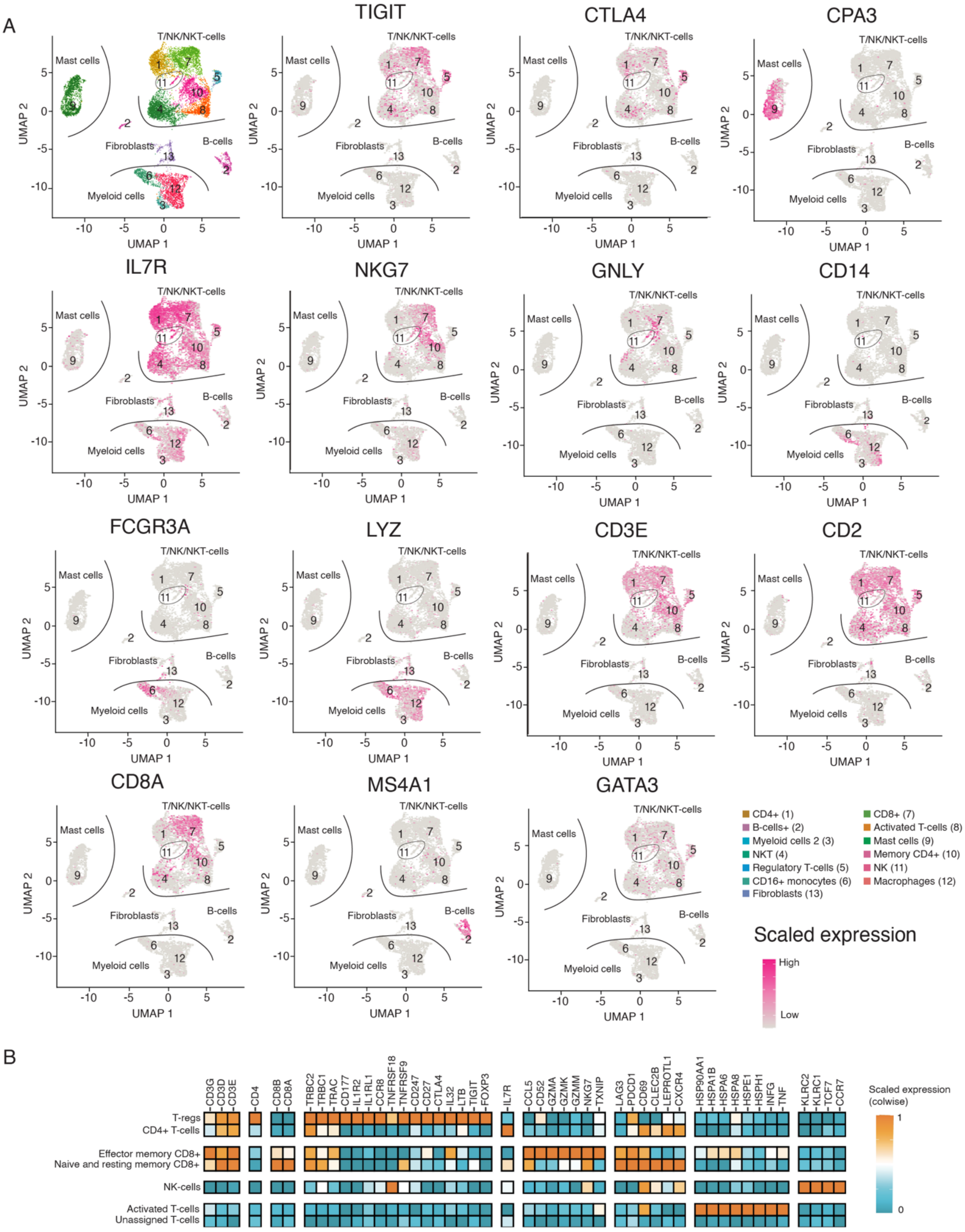

**Supplementary Figure 4. Feature map and T-cells markers for cluster identification in single cell sequencing. a,** A feature map representation of the main markers used for cluster identification in the single cell sequencing data-analysis. **b,** The relative (scaled) expression of main T-cell markers used for cluster identification in the single cell sequencing data-analysis.

**Supplementary Figure 5.**

A

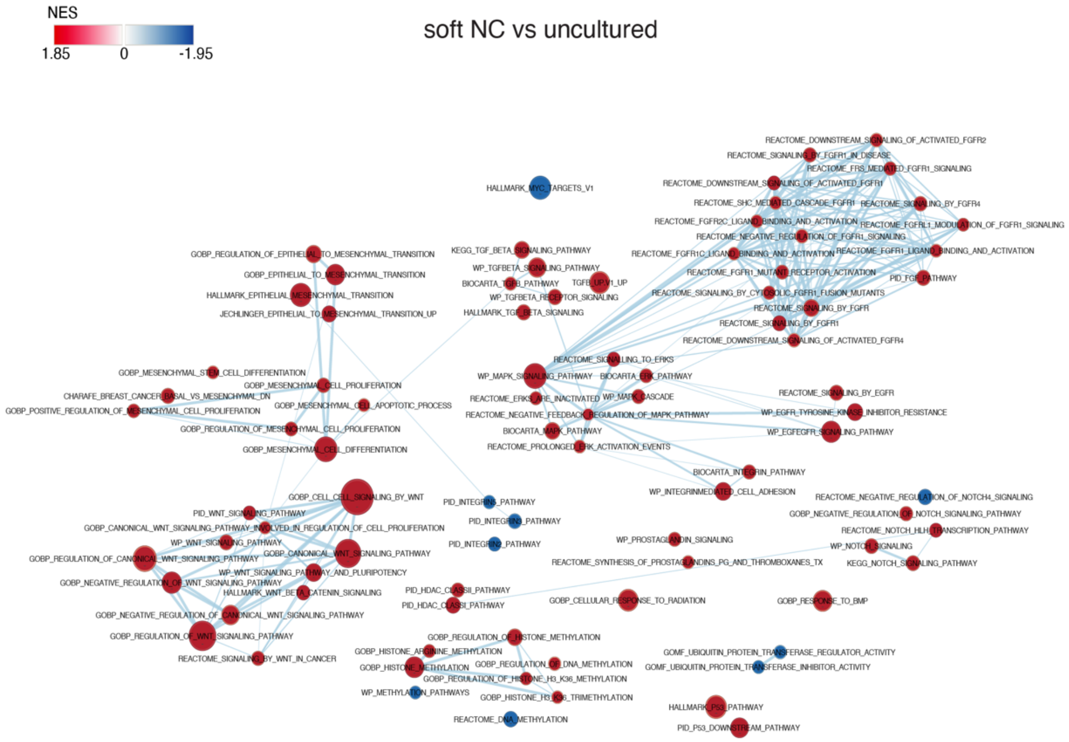


B

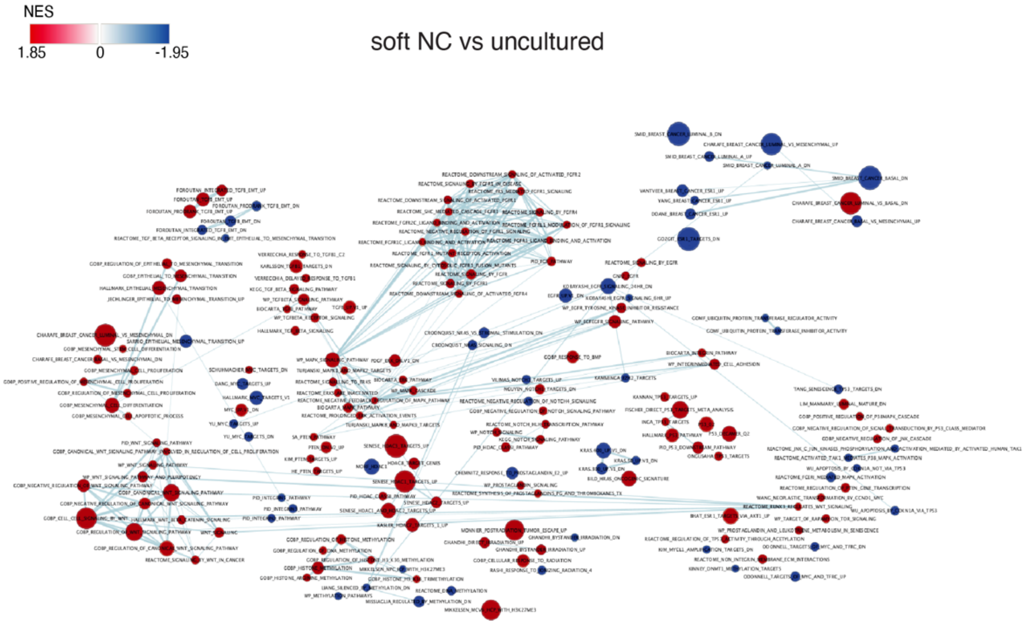

**Supplementary Figure 5. Enrichment map of soft NC enriched pathways. a,** The figure shows all significantly differentially expressed gene sets (FDR<0.2) between the soft nanocellulose (NC) gel and the uncultured sample visualized in Fig 3A. Gene sets were from the following collections KEGG, BIOCARTA, GOPB, HALLMARK, GO, and PID. **b,** The figure shows all significantly differentially expressed gene sets (FDR<0.2) between the soft NC gel and the uncultured samples.

**Supplementary Figure 6.**

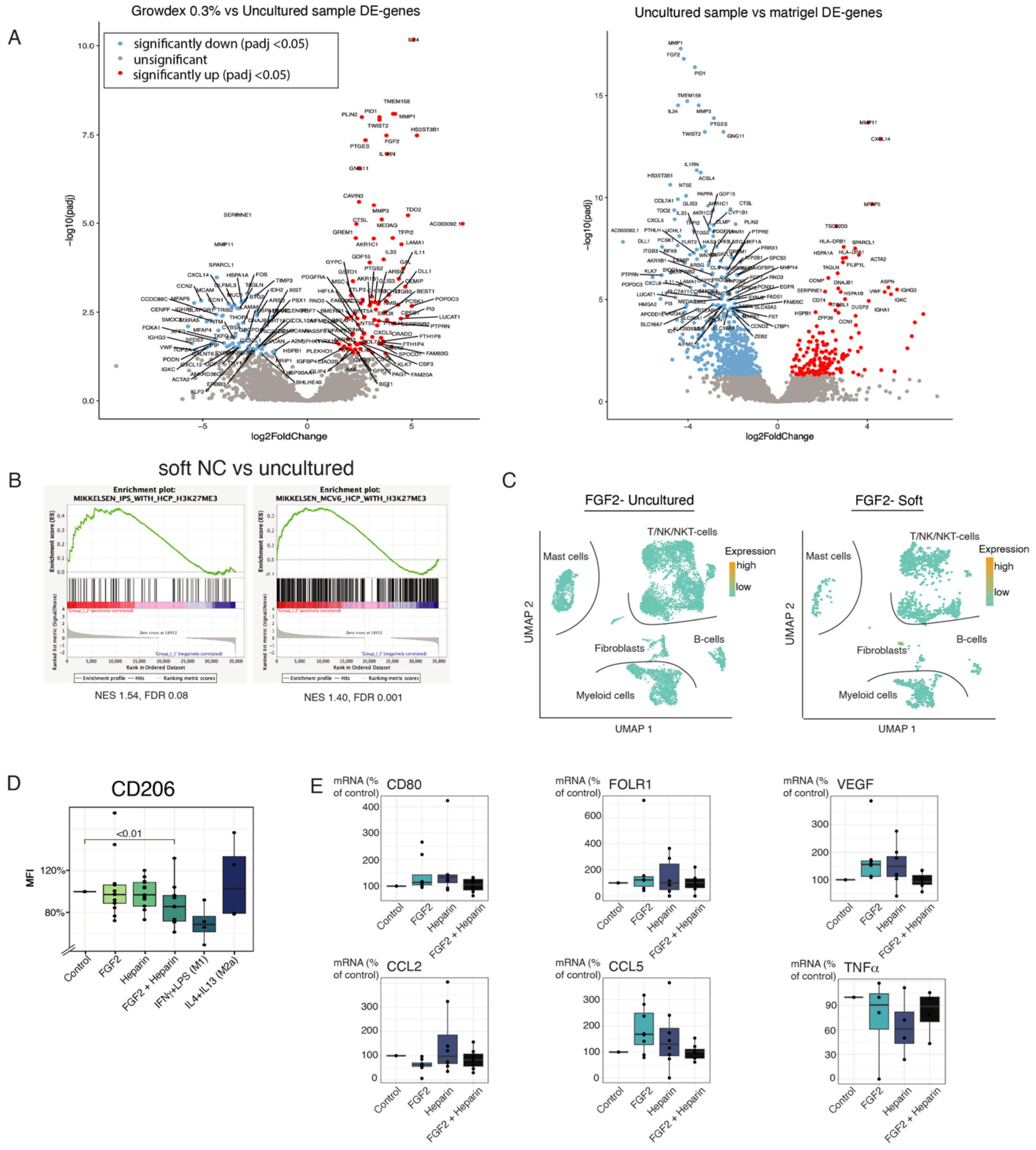


**Supplementary Figure 6. a,** Top differentially expressed genes in the uncultured samples, samples grown in the soft nanocellulose (NC) gel, and in the Matrigel. **b,** Gene sets representing the upregulation of EZH2 in the soft NC gel. The gene sets were significantly upregulated in the soft NC gel grown samples in comparison to the uncultured sample (n=5 for the uncultured sample and n=4 for the soft NC gel cultures.) **c,** Feature plot representation of *fibroblast growth factor 2* (*FGF2*) expression in the uncultured sample and in the soft NC gel (single cell RNA sequencing).

**d,** Median expression of CD206 in M0 macrophages cultured together with FGF2, heparin, and heparin + FGF2 for 24 hours and compared with lipopolysaccharide (LPS) + interferon gamma (IFNγ) (M1) and IL-4 + IL-13 (M2) treated macrophages (M1/M2 n=4, n=12). **e,** qRT-PCR analysis with 6 M1 and M2 related primers from the same conditions.

**Supplementary Figure 7.**

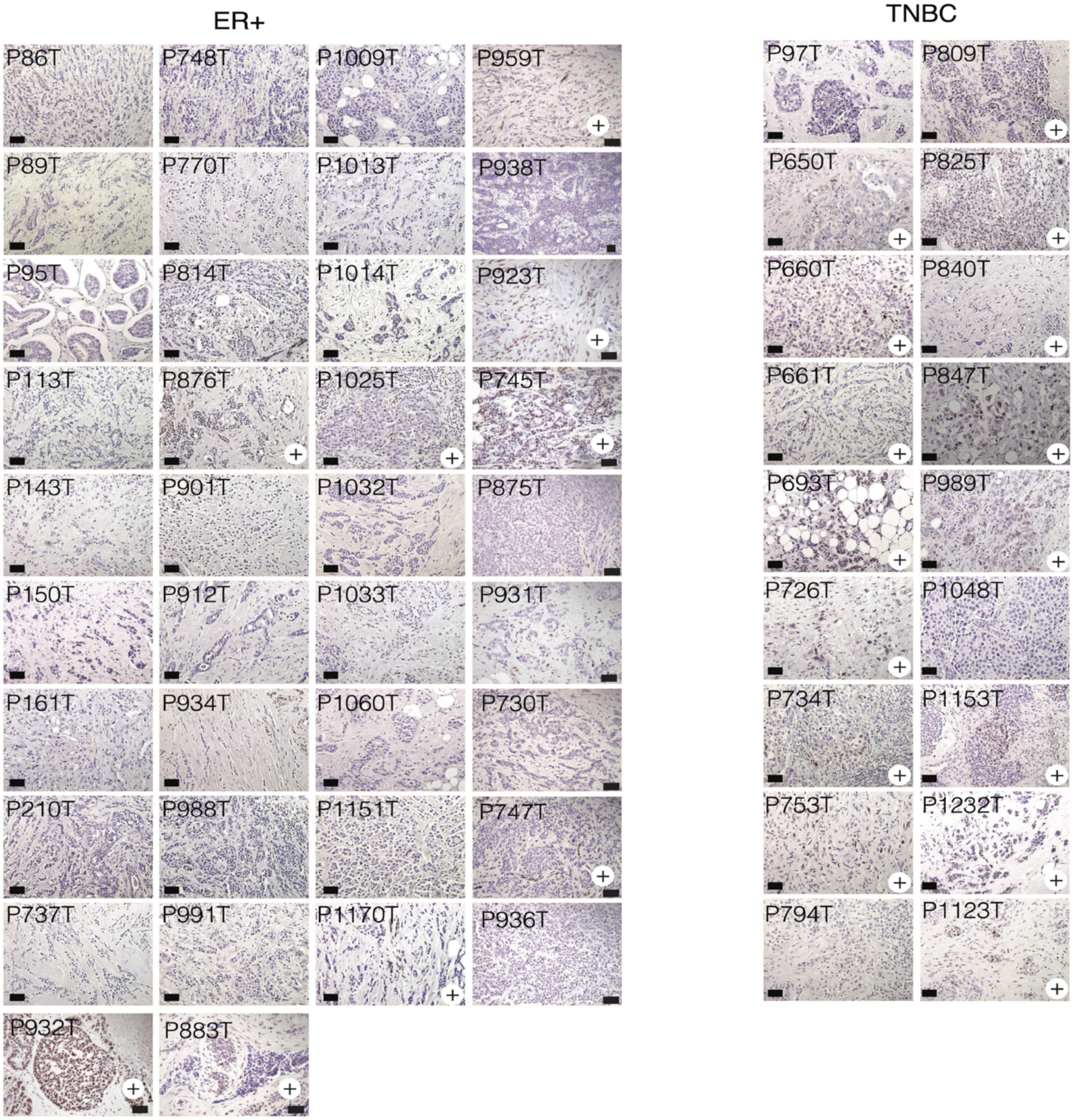


**Supplementary Figure 7. FGF2 expression in breast cancer samples.** Immunohistochemical staining of fibroblast growth factor 2 (FGF2) from 45 breast cancer samples with either estrogen receptor (ER+) or triple negative breast cancer (TNBC) subtype. Plus-sign indicates the positive samples (+). Scale bars represent 100µm.

**Supplementary Figure 8.**

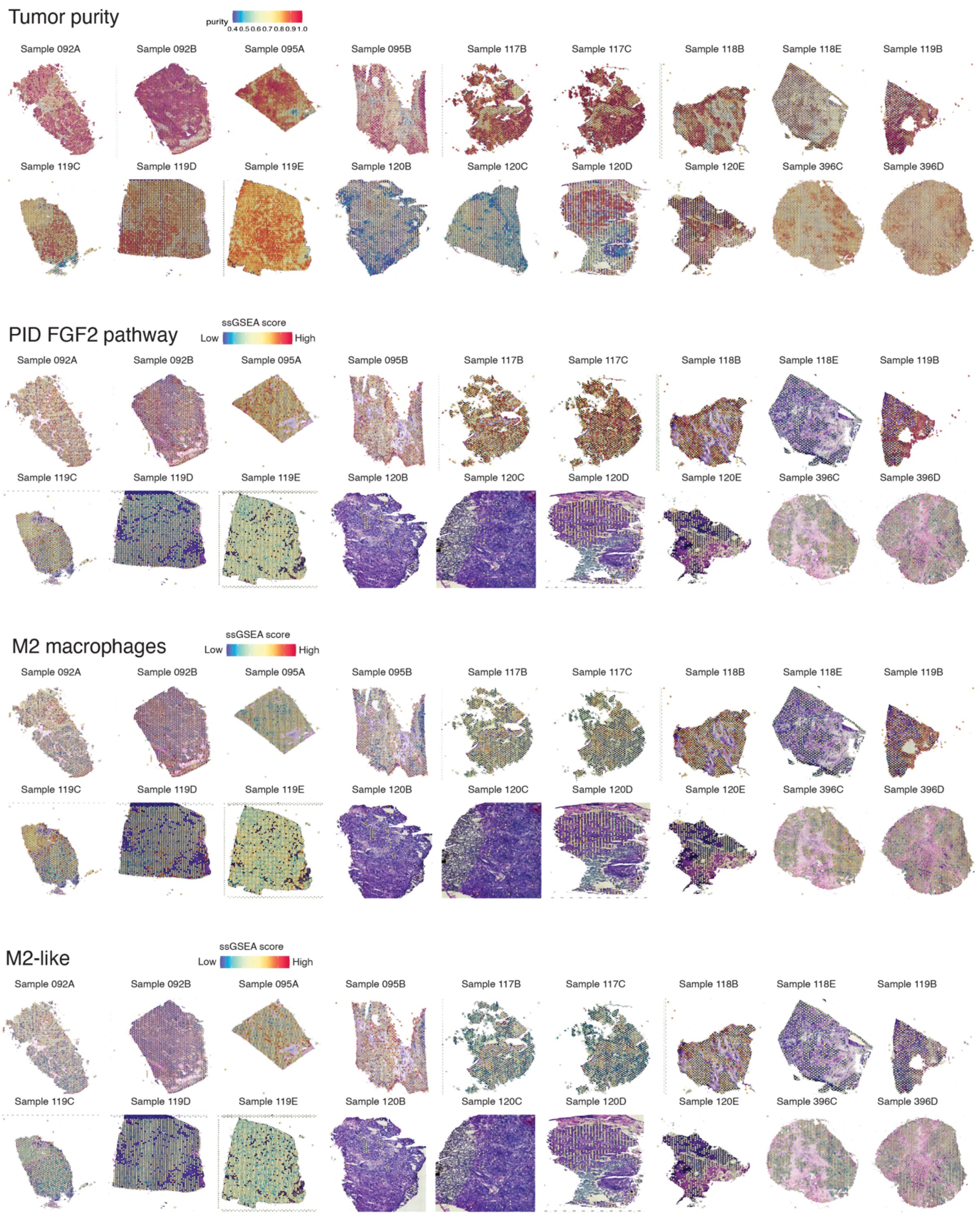

**Supplementary Figure 9.**

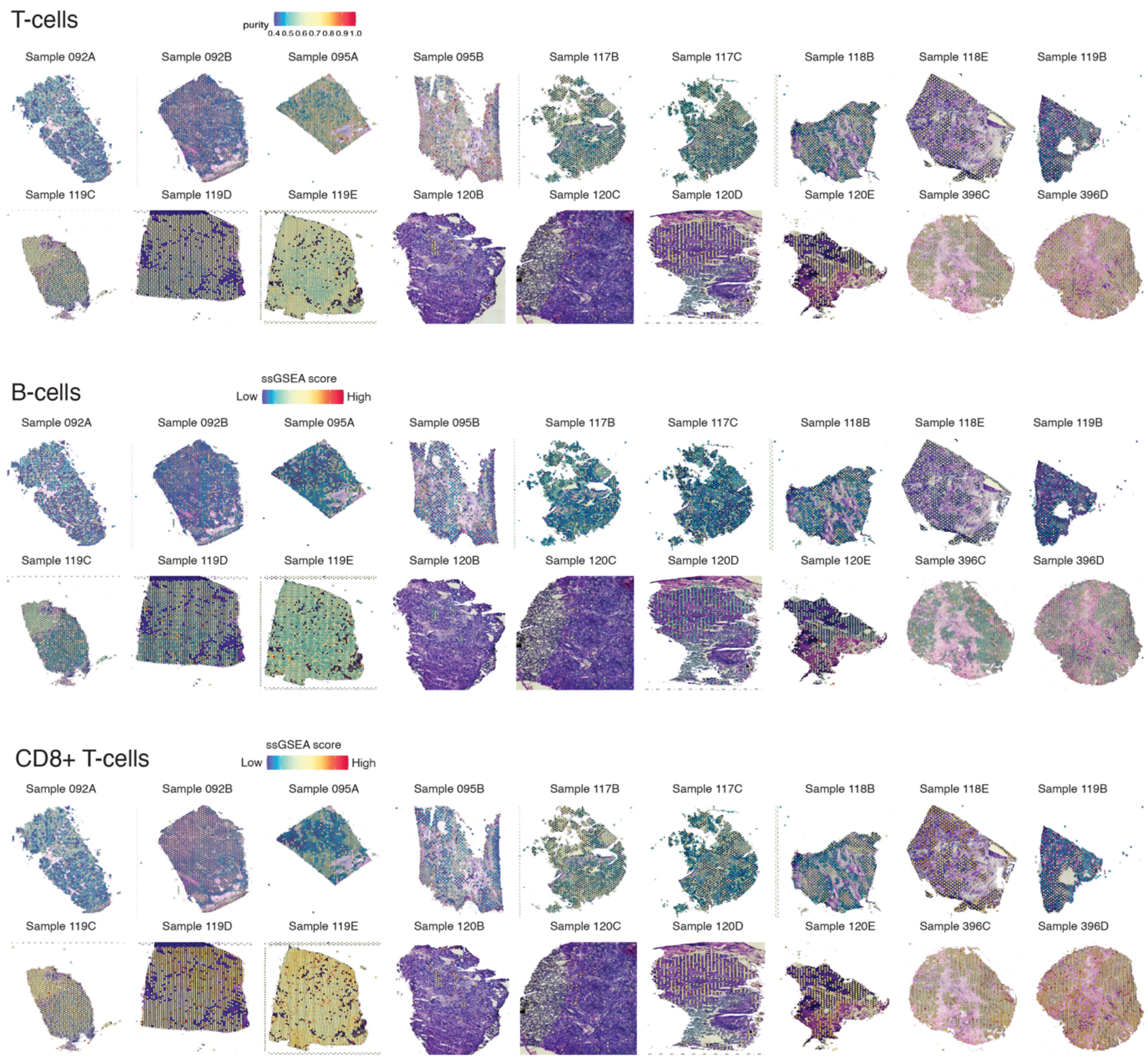


**Supplementary Figure 8-9. Spatial transcriptomics.** The spatial expression of PID_FGF_Pathway, as well as T-, B-cell, and macrophage related gene sets.

## Supplementary Figure 10.

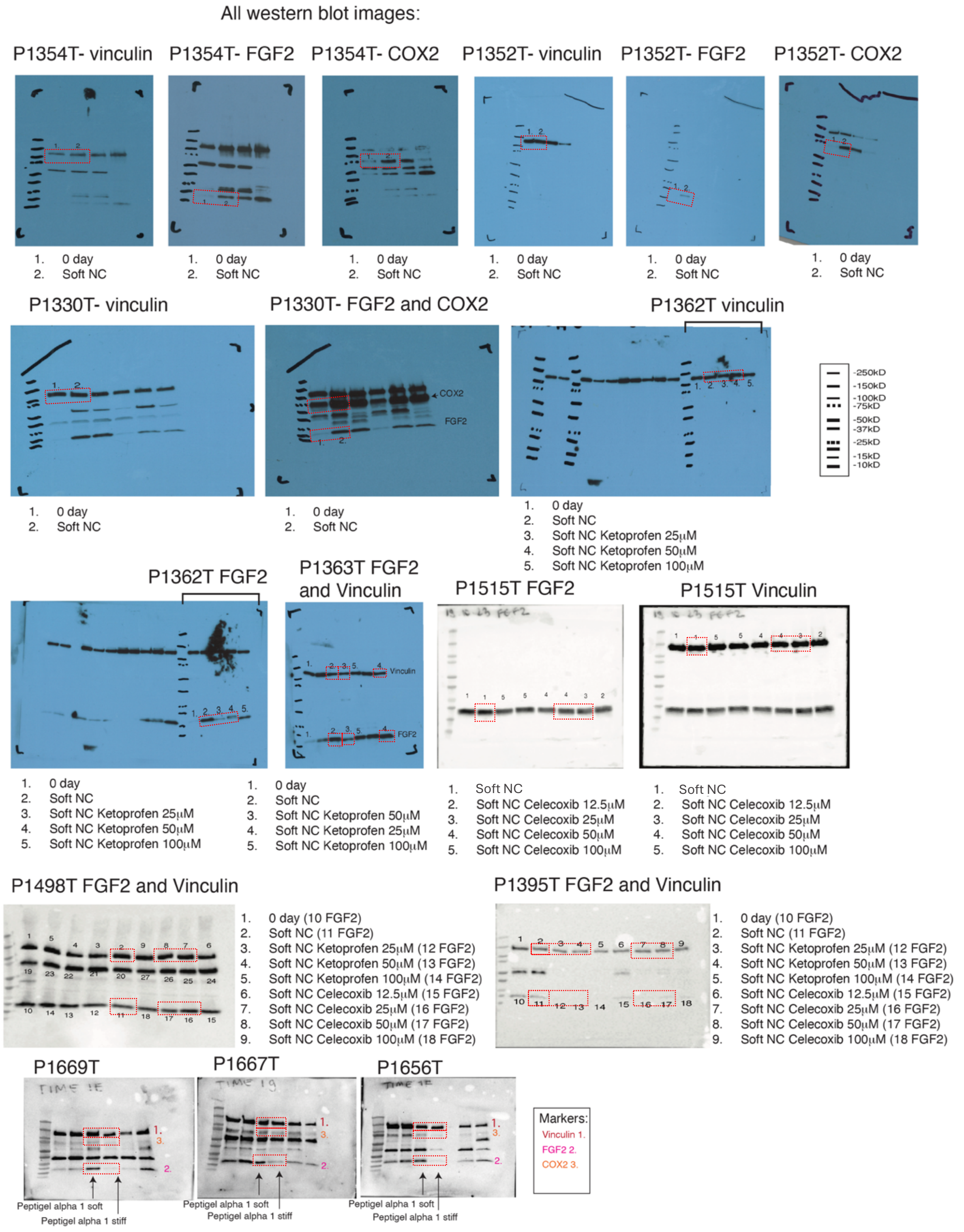


**Supplementary Figure 10. Original western blots.** Raw images of all western blots.

**Supplementary Figure 11.**

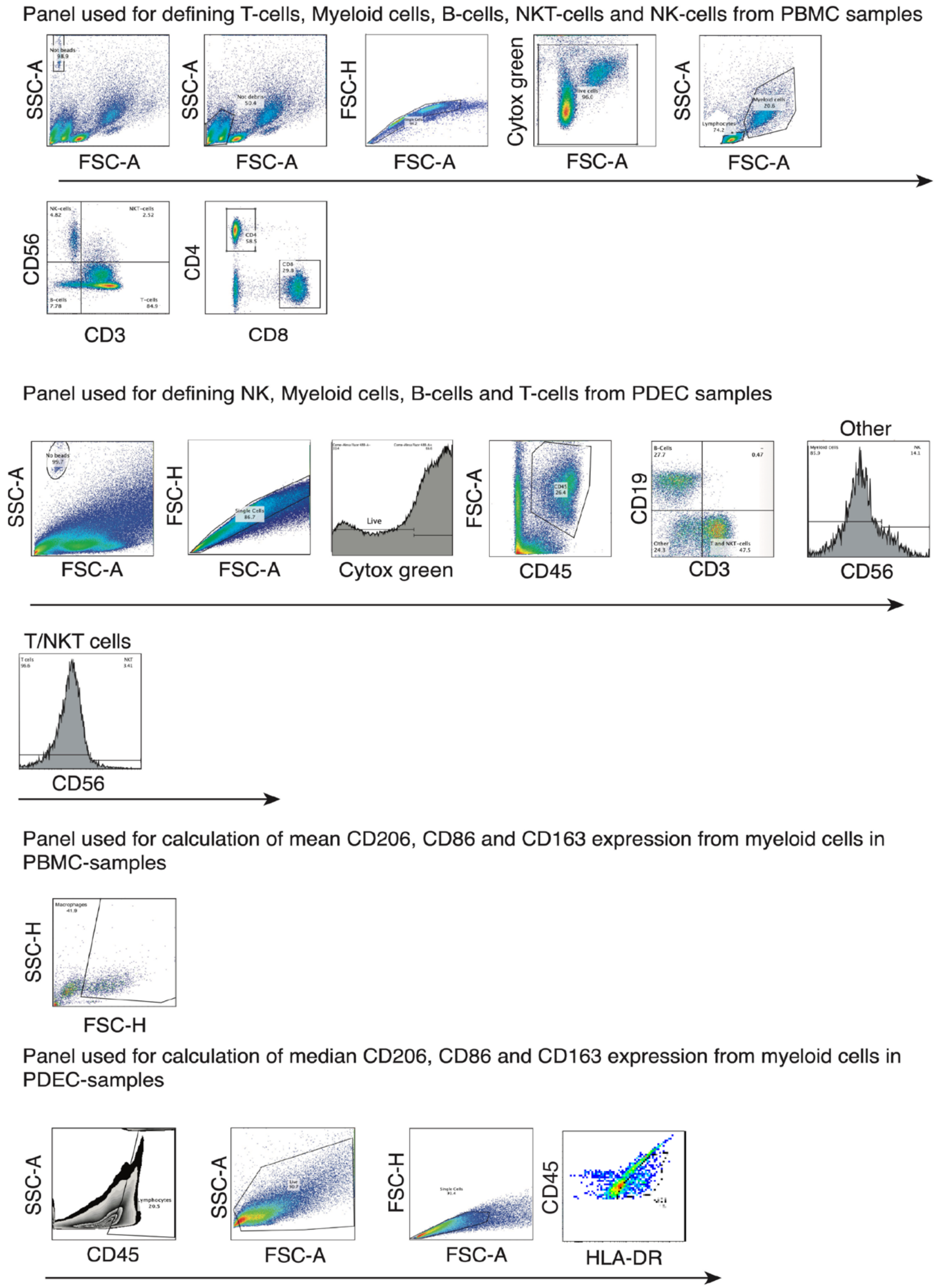


**Supplementary Figure 11. Flow cytometry gating.** Flow cytometry panels used for defining the number of immune cells and relative macrophage phenotypes in different matrices.

**Supplementary Figure 12.**

P959T

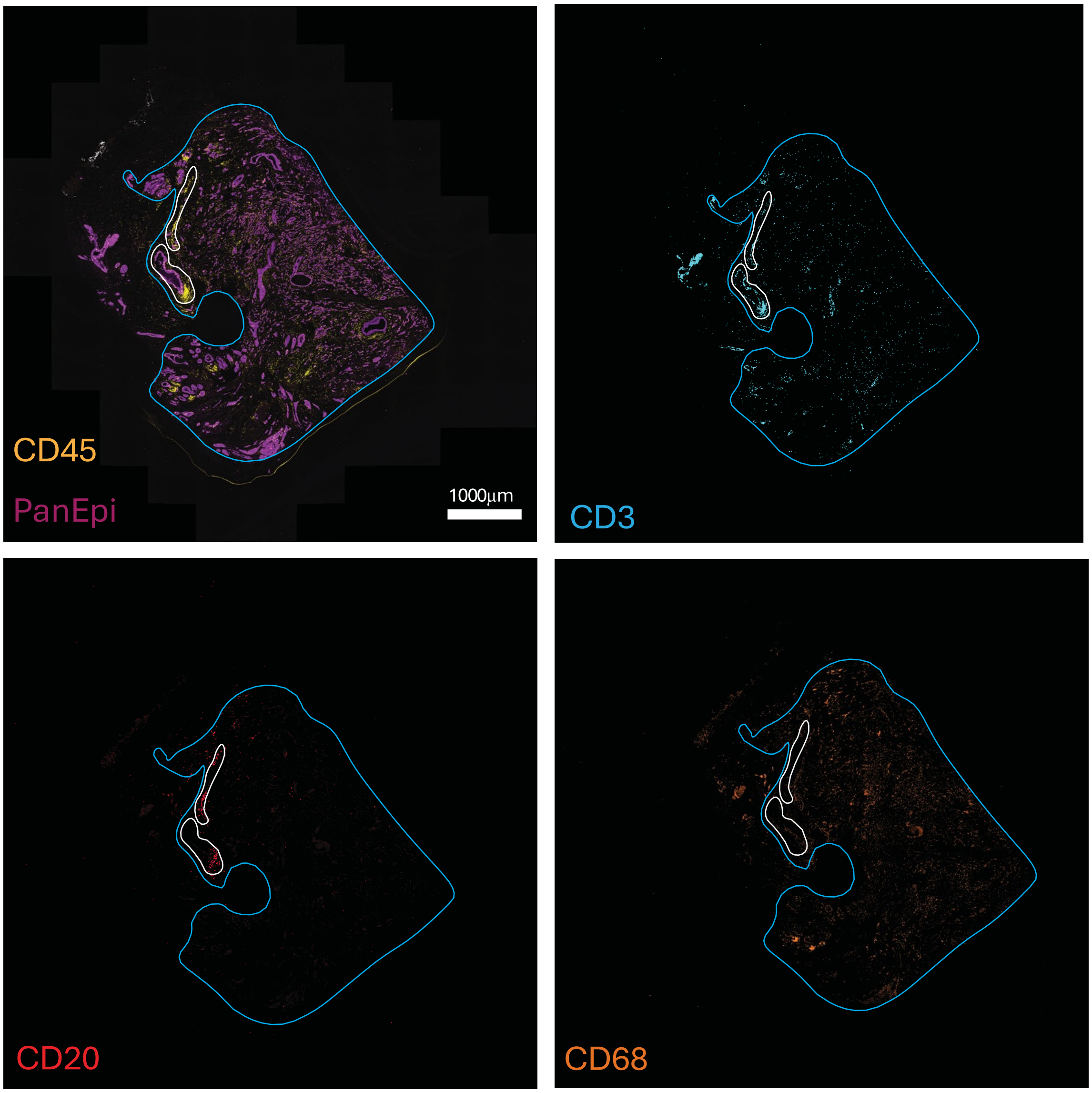


FGF2 expression

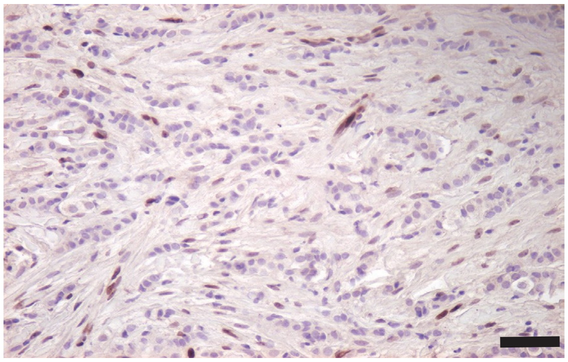

P938T

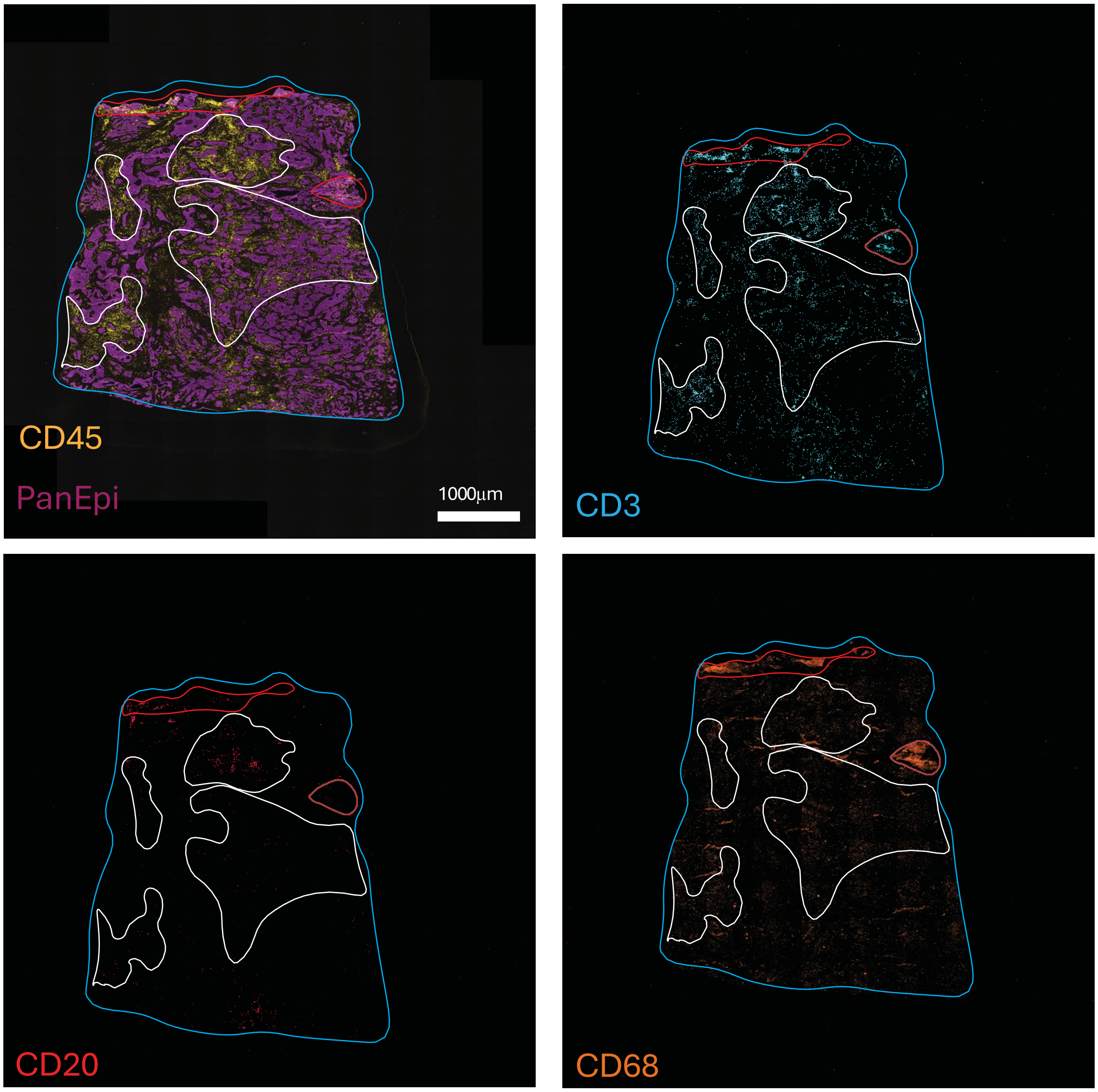


FGF2 expression

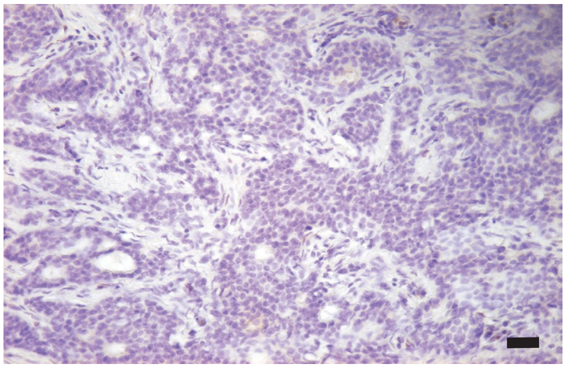

P923T

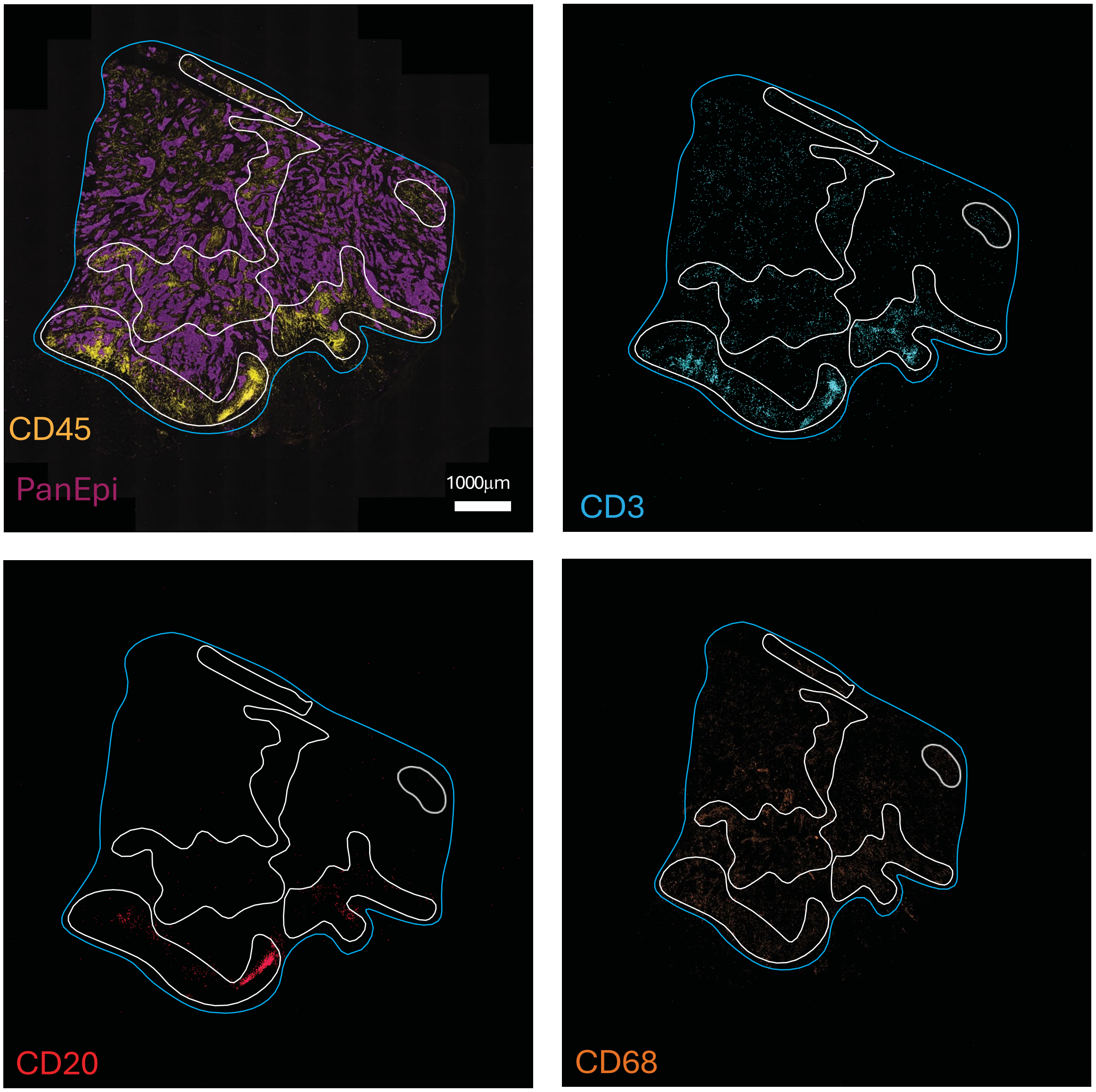


FGF2 expression

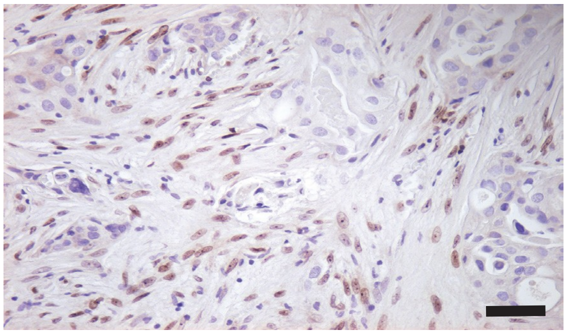

P745T

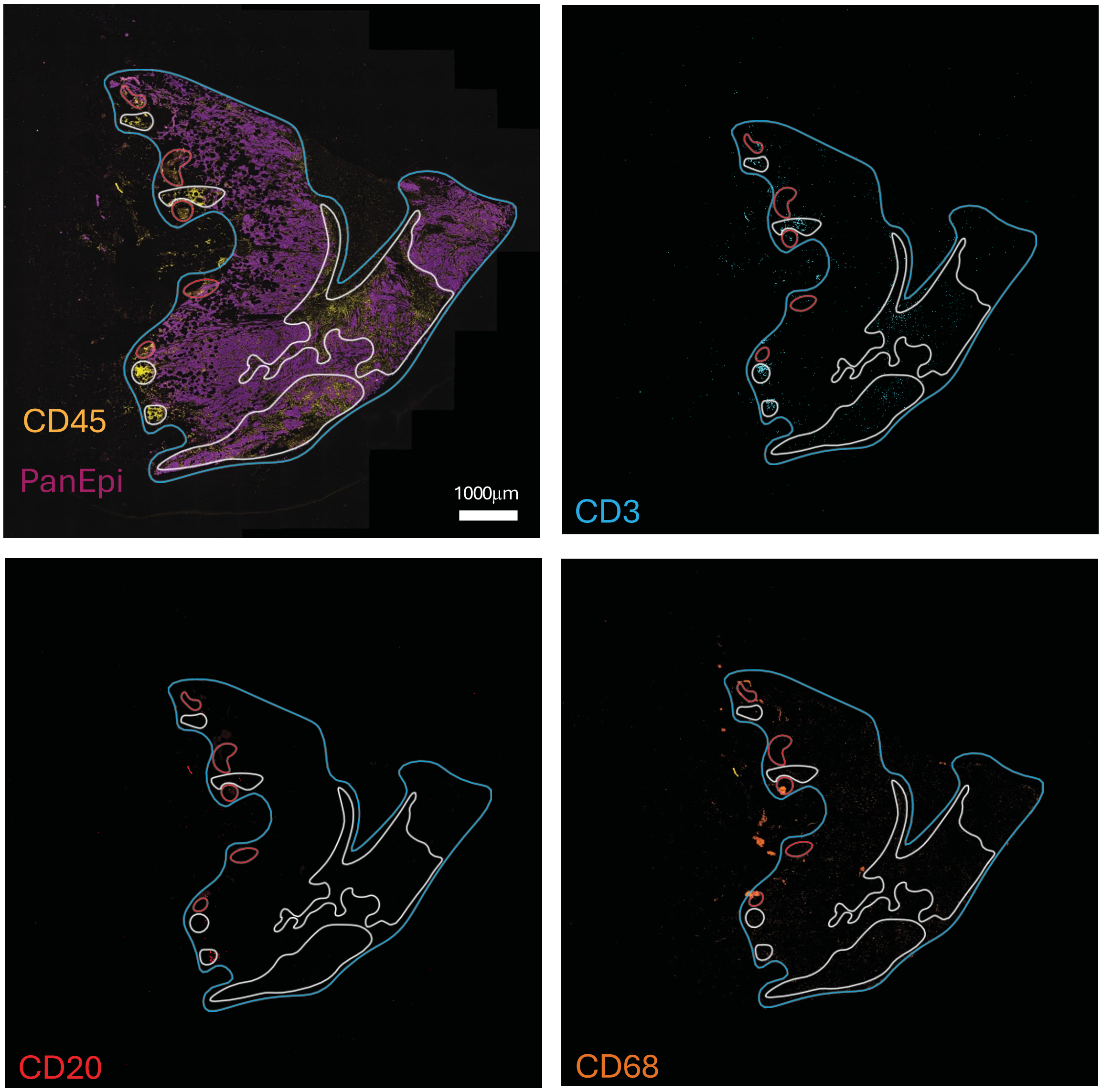


FGF2 expression

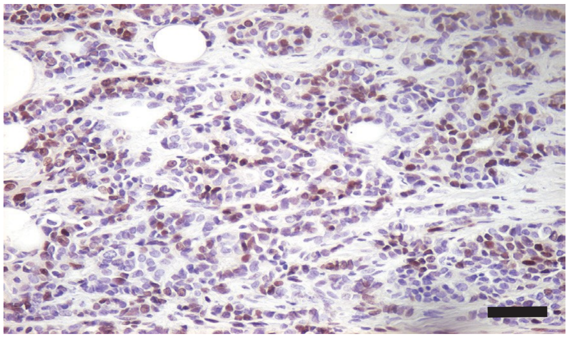

P883T

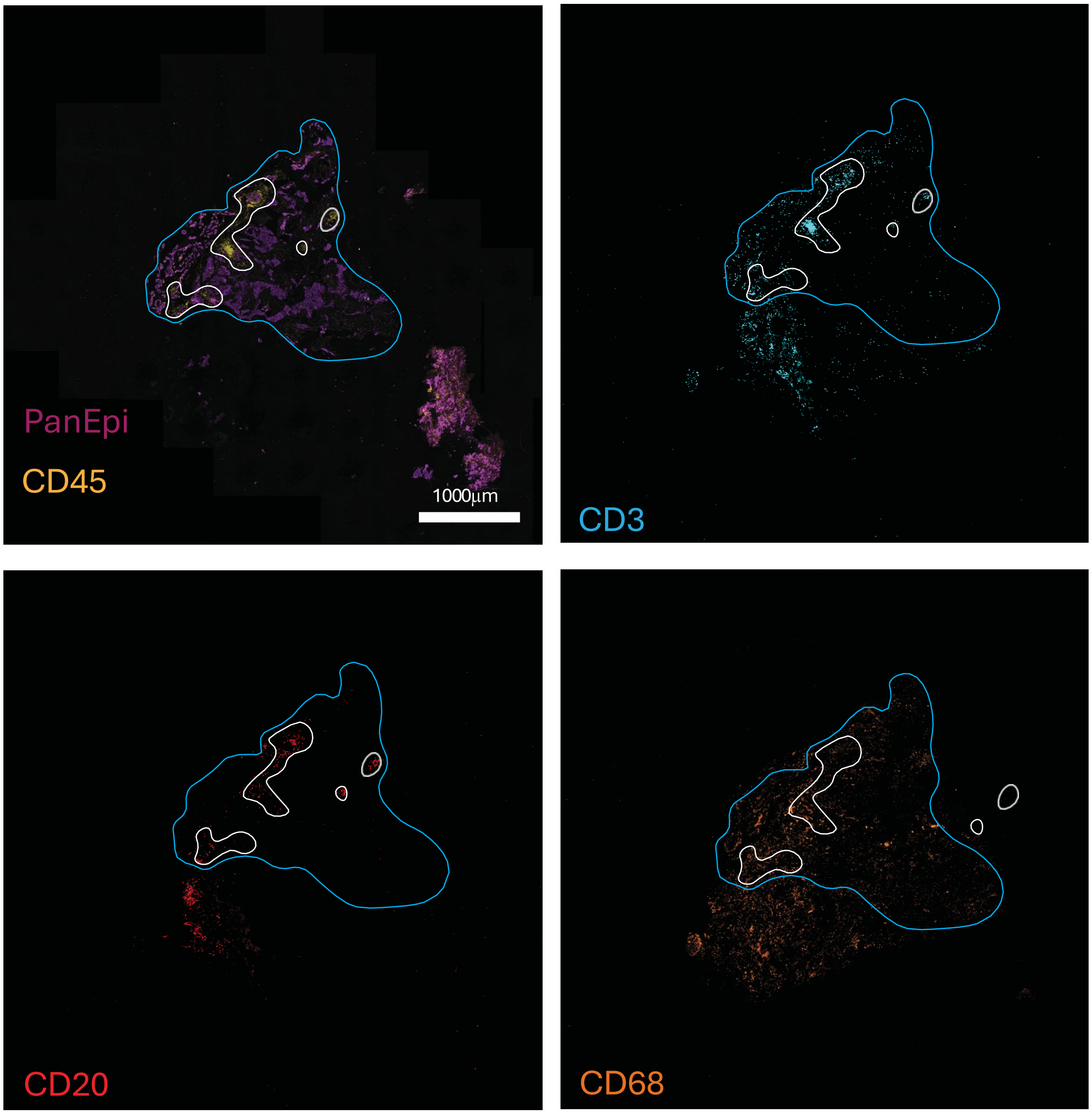


FGF2 expression

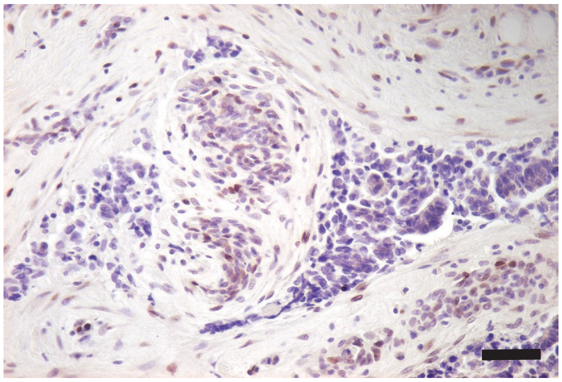

P932T

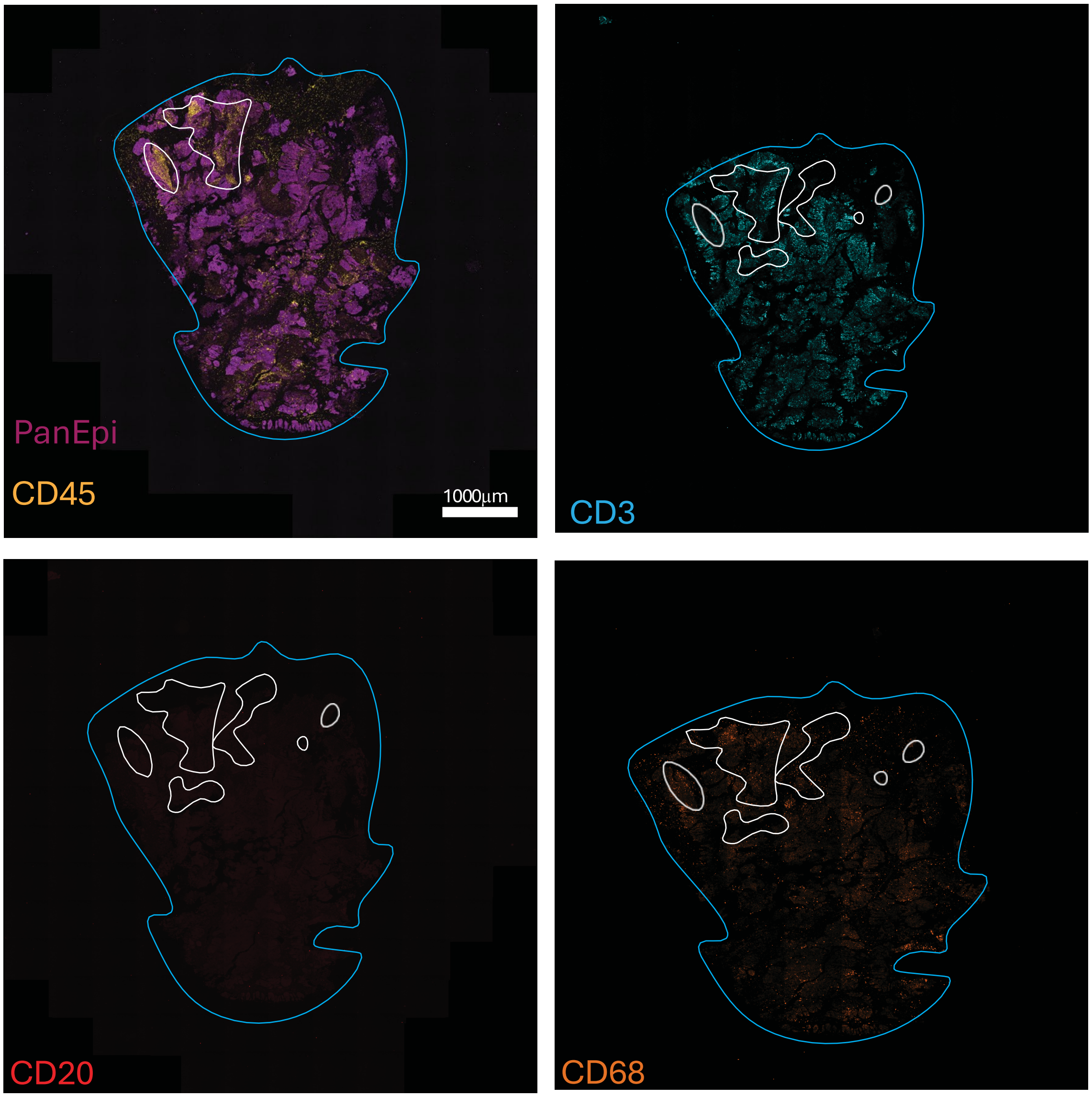


FGF2 expression

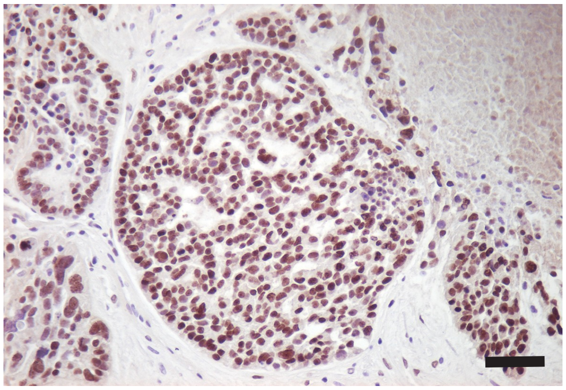

P936T

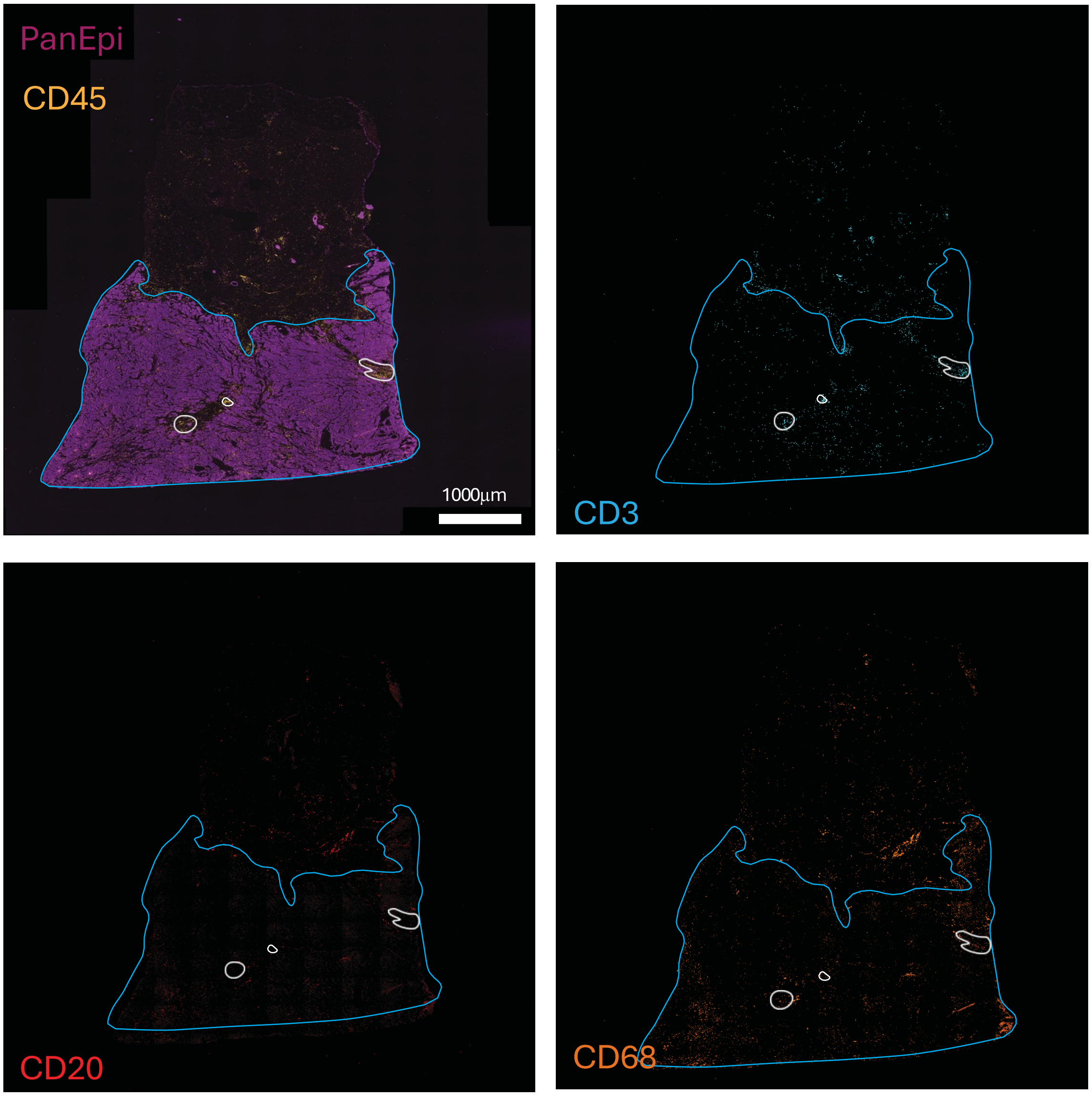


FGF2 expression

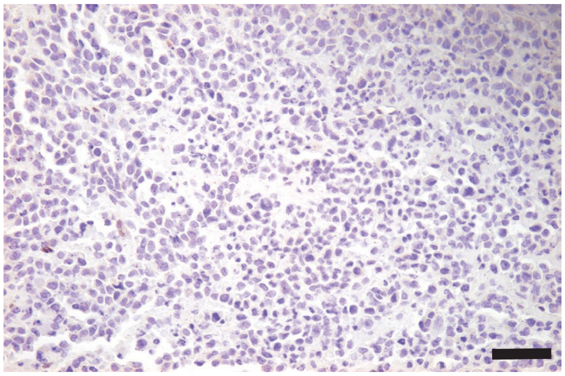

P747T

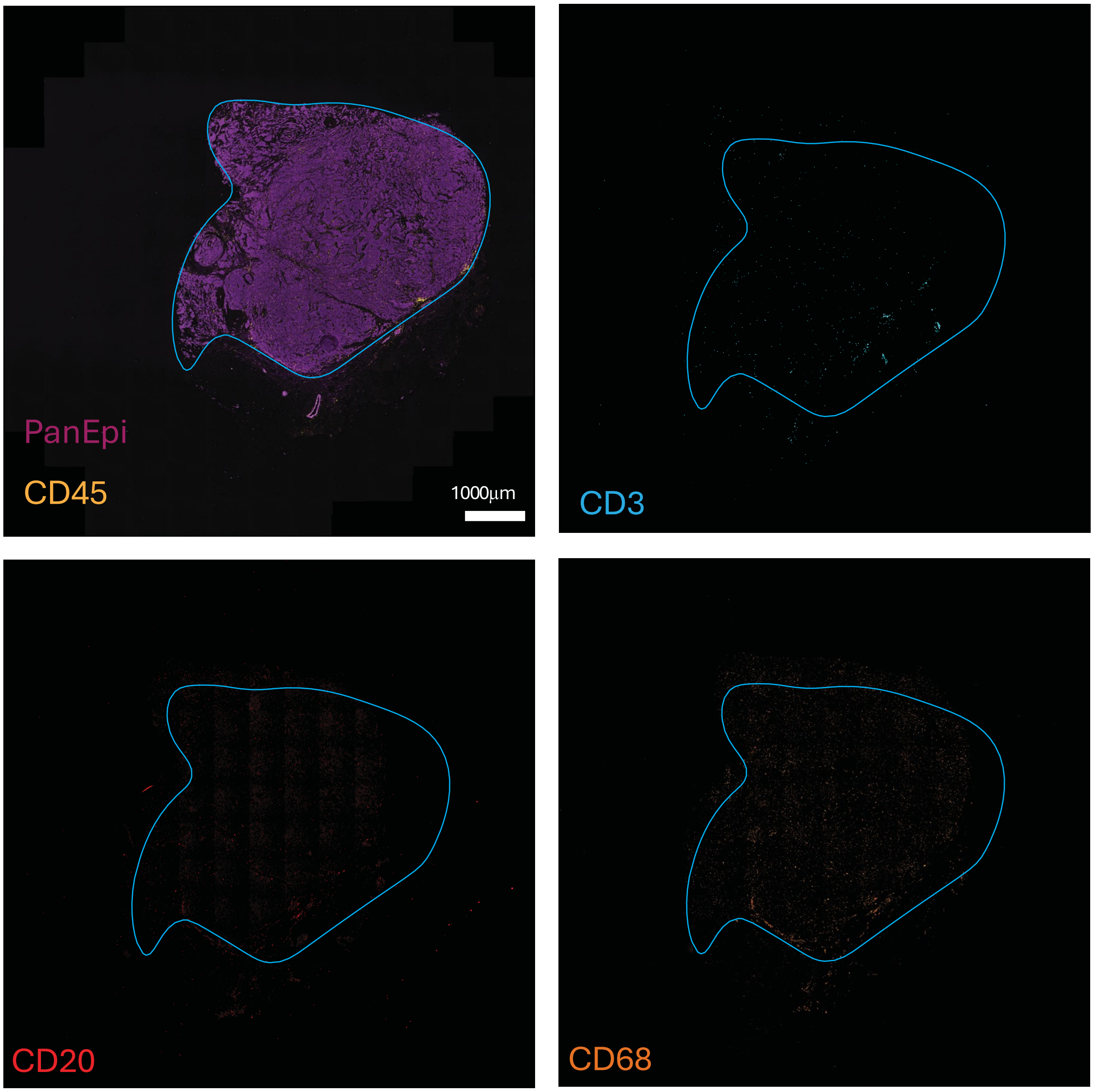


FGF2 expression

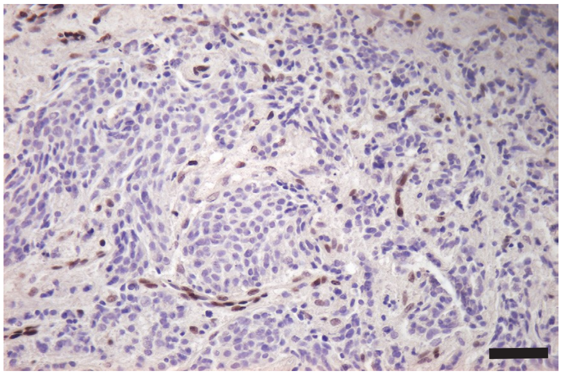

P730T

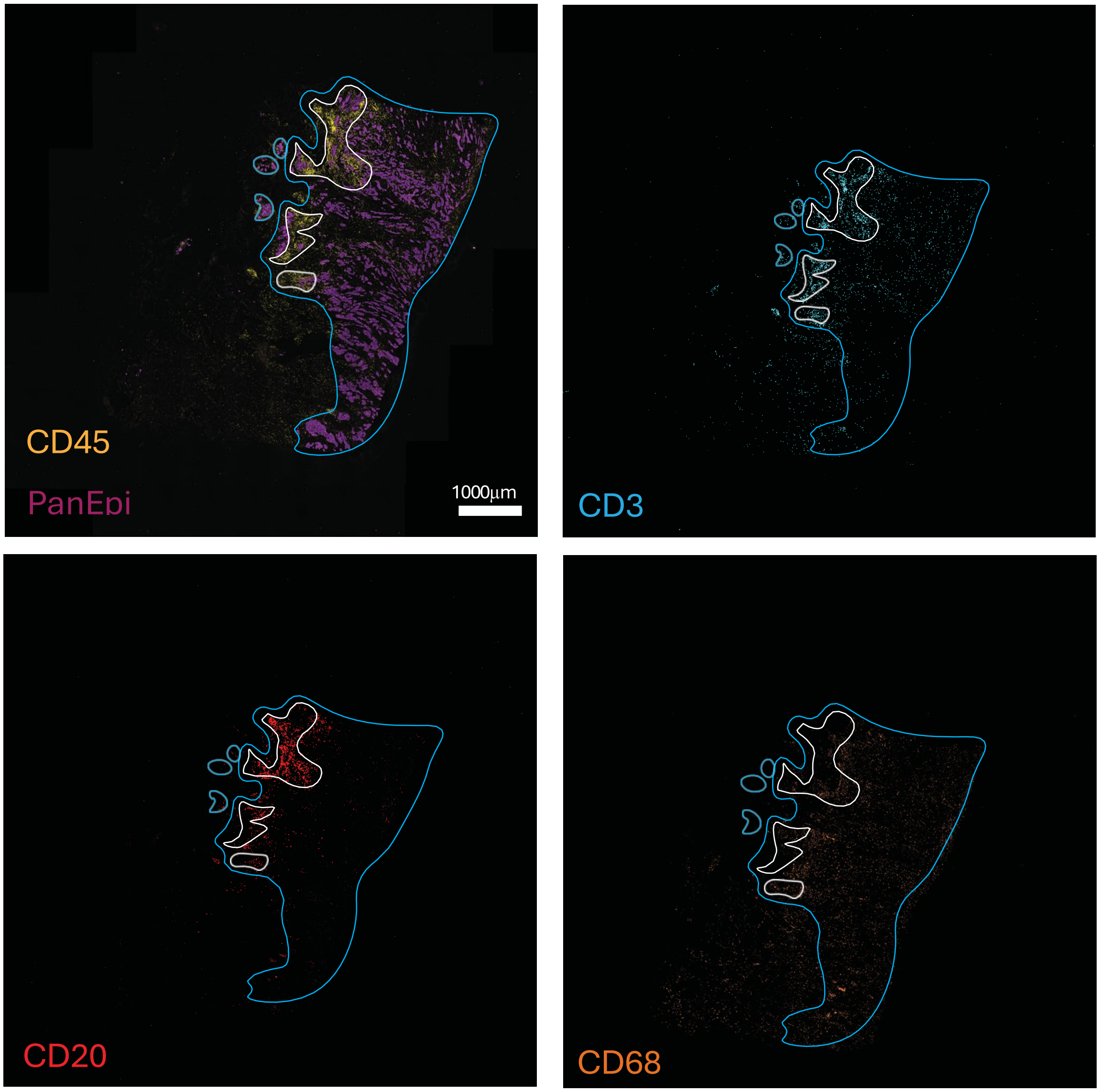


FGF2 expression

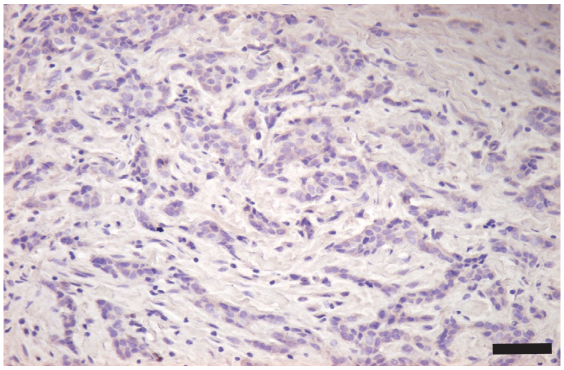

P931T

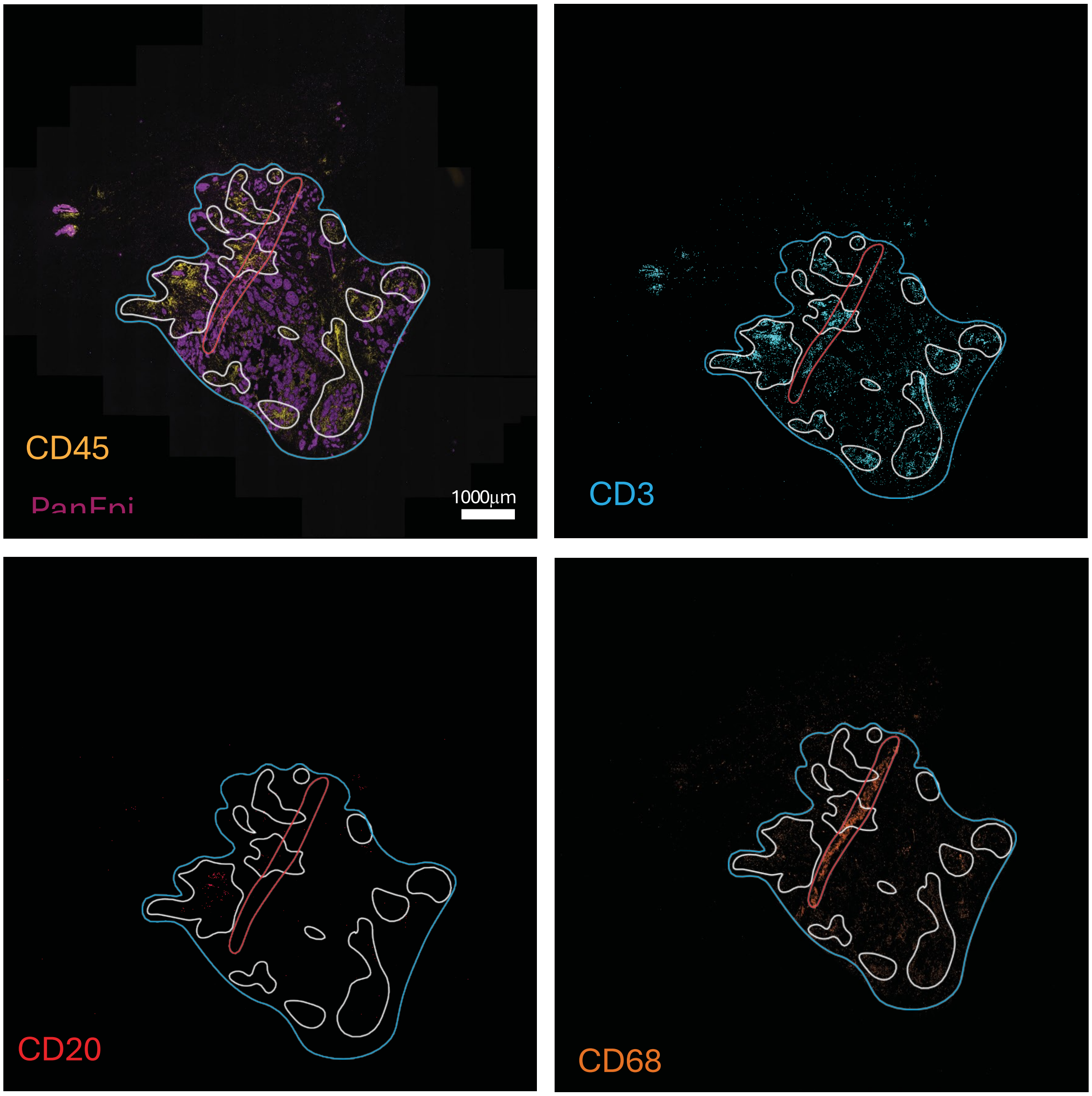


FGF2 expression

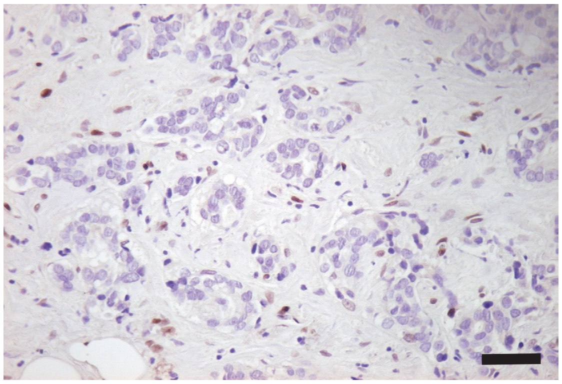

P875T

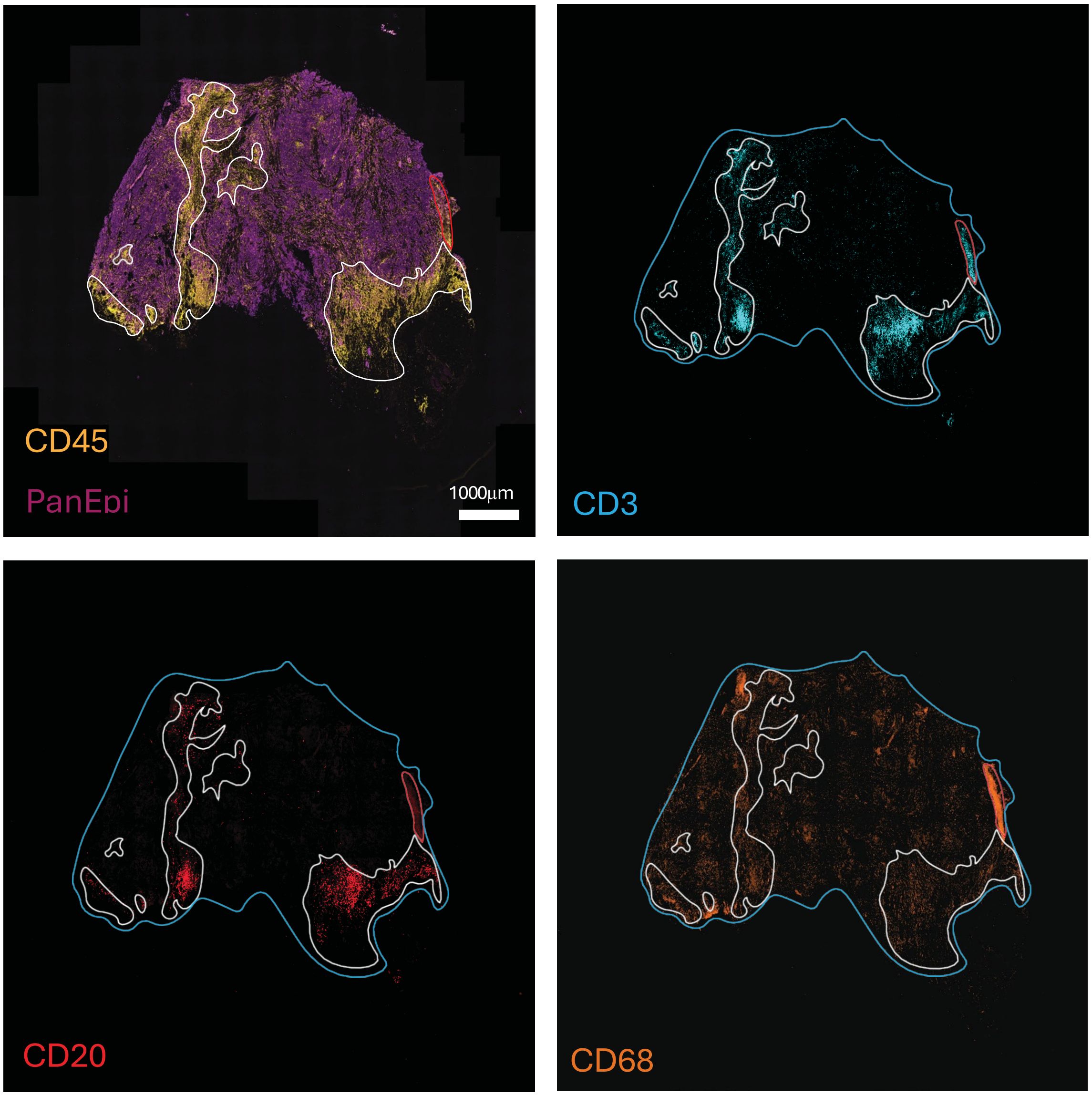


FGF2 expression

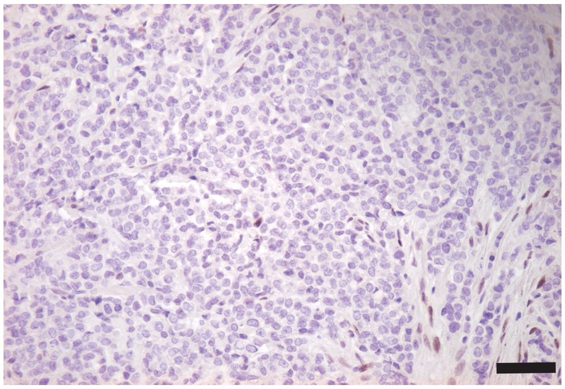

**Supplementary Figure 12. Multiplex immunohistochemistry.** Full size images of the multiplex images from samples stained with CD45, EPCAM, CD3, CD20, and CD68. CD45-rich areas are encircled with white lines. The red line encircles a wrinkle in the section. Fibroblast growth factor 2 (FGF2) staining from the same samples. Scale bar in the images is 100 μm.

**Supplementary Table 1**

| **Flow cytometry antibodies** | |
|---|---|
| **Antibody-conjugate** | **Clone & Manufacturer** |
| CD3-PerCP-Cy5.5 | Clone SK7; BD Biosciences 332771 1:160 |
| CD4-PE-Cy7 | Clone RPA-T4, BD Biosciences 560649 1:160 |
| CD8-BV510 | Clone SK1, BD Biosciences 563919 1:160 |
| CD19-PE-CF594 | Clone HIB19 (RUO), BD Biosciences 562321 1:160 |
| CD69-APC | Clone L78 (ASR), BD Biosciences 654663 1:160 |
| CD56-BV421 | Clone NCAM16.2, BD Biosciences 562751 1:160 |

| | |
|---|---|
| CD3-PerCP-Cy5.5 | Clone SK7; BD Biosciences 332771 1:160 |
| CD56-BV421 | Clone NCAM16.2, BD Biosciences 562751 1:160 |
| CD14-APC | Clone M5E2 (RUO), BD Biosciences 555399 1:160 |

| | |
|---|---|
| CD16-PE | Clone B73.1, BD Biosciences 561313<br><br>1:160 |
| CD80-BV510 | Clone L307.4, BD Biosciences 563084<br><br>1:160 |
| CD86-PE-Cy7 | Clone 2331, BD Biosciences 561128<br><br>1:160 |
| CD45-APC-H7 | Clone 2D1, BD Biosciences 560178 1:160 |
| CD11c-FITC | Clone 3.9, BioLegend 1:160 |
| CD206-Alexa Fluor 700 | Clone 15-2, BioLegend 1:160 |
| CD163-BV650 | Clone GHI/61, BD Biosciences 563888<br><br>1:160 |
| Sytox green A | Invitrogen™ S34860 1:160 |
| CD103-PerCP-Cy5.5 | Clone Ber-ACT8 BioLegend 350225 1:160 |
| TIGIT-APC | Clone A15153C BioLegend 372705 1:160 |
| CCR7-APC-Cy7 | Clone G043H7 BioLegend 2D1 1:160 |
| CD45-BV421 | Clone 2D1 BioLegend 368521 1:160 |
| PD1-BV510 | Clone NAT105 BioLegend 1:160 |
| CD39-BV605 | Clone A1 BioLegend 328235 1:160 |
| CD8-BV711 | Clone RPAT8 BioLegend 301043 1:160 |
| CD45RA-BV785 | Clone HI100 BioLegend 304139 1:160 |
| | |

| | |
|---|---|
| **IF-staining** | |
| ki67 | Abcam, ab15588 |
| CC3 | Cell signaling, # 9661 |
| F-actin | Thermo Fisher Scientific, AlexaFluor 546, A22283 |
| **Western blot analysis** | |
| Vinculin | ab129002 Abcam, 1:5000-1:10 000 |
| FGF2 | Abcam ab208687 1:1000-1:5000 |
| Cox2 | Cell signaling #12282 1:1000 |
| | |
| **Multiplex staining** | |
| CD3 | CD3, Thermo MA14482; 1:100 |
| CD20 | CD20, Thermo MS-340; 1:50 |
| CD68 | Abcam ab955; 1:50 |
| CD45 | CD45, CST13917 1:100 |
| PanCK | Abcam ab7753 1:150 |
| | |
| **IHC staining** | |
| FGF2 | Abcam ab208687 1:1000-1:5000 |

| **Live imaging** | |
|---|---|
| CD45 | VioBright R676 1:100 |

| QRT-PCR primers | |
|---|---|
| Sequence | Target and source |
| CAGCCAGATGCAATCAATGCC | CCL2 forward |
| TGGAATCCTGAACCCACTTCT | CCL2 reverse |
| CCTCTGGAGTCACTCATACAAGG | FOLR2 forward |
| CCTGGGCTGAATCAAACCAC | FOLR2 reverse |
| CCTCTCTCTAATCAGCCCTCTG | TNFα forward |
| GAGGACCTGGGAGTAGATGAG | TNFα reverse |
| AGGGCAGAATCATCACGAAGT | VEGF forward |
| AGGGTCTCGATTGGATGGCA | VEGF reverse |
| CCAGCAGTCGTCTTTGTCAC | CCL5 forward |
| CTCTGGGTTGGCACACACTT | CCL5 reverse |

**Supplementary Table 1.** Antibodies, concentrations and dilutions used in the study.
