# Supplementary material for: Soft matrix promotes immunosuppression in tumor-resident immune cells via COX-FGF2 signaling
Source: Nat Commun. 2025 May 27;16:4908. doi: 10.1038/s41467-025-60092-x (PMC12116891; doi:10.1038/s41467-025-60092-x)
Supplement: Supplementary file 2 — Description of Additional Supplementary Files [file 41467_2025_60092_MOESM2_ESM.pdf]

**Description of Additional Supplementary Files**

**Supplementary Movie 1:** The time-lapse imaging of patient derived explant cultures (PDEC) after 6 days of ex vivo culture. The movie demonstrates the motility of tumor infiltrating lymphocytes (TIL) in the soft 0.3% nanocellulose (GrowDex/GD) cultures. The TILs are stained with CD45+ VioBright for live imaging. The sample is tumor adjacent normal tissue.

**Supplementary Movie 2:** The time-lapse imaging of patient derived explant cultures (PDEC) after 6 days of ex vivo culture. The movie demonstrates the motility of tumor infiltrating lymphocytes (TIL) in the soft 0.3% nanocellulose (GrowDex/GD) cultures. The TILs are stained with CD45+ VioBright for live imaging. The sample is tumor sample.

**Supplementary Movie 3:** The time-lapse imaging of patient derived explant cultures (PDEC) after 6 days of ex vivo culture. The movie demonstrates the motility of tumor infiltrating lymphocytes (TIL) in the soft 0.3% nanocellulose (GrowDex/GD) cultures. The TILs are stained with CD45+ VioBright for live imaging. The sample is tumor sample.

**Supplementary Movie 4:** The time-lapse imaging of patient derived explant cultures (PDEC) after 6 days of ex vivo culture. The movie demonstrates the motility of tumor infiltrating lymphocytes (TIL) in the soft 1% nanocellulose (GrowDex/GD) cultures. The TILs are stained with CD45+ VioBright for live imaging. The sample is tumor adjacent normal tissue.

**Supplementary Movie 5:** The time-lapse imaging of patient derived explant cultures (PDEC) after 6 days of ex vivo culture. The movie demonstrates the motility of tumor infiltrating lymphocytes (TIL) in the soft 1% nanocellulose (GrowDex/GD) cultures. The TILs are stained with CD45+ VioBright for live imaging. The sample is tumor sample.

**Supplementary Movie 6:** The time-lapse imaging of patient derived explant cultures (PDEC) after 6 days of ex vivo culture. The movie demonstrates the motility of tumor infiltrating lymphocytes (TIL) in the soft 1% nanocellulose (GrowDex/GD) cultures. The TILs are stained with CD45+ VioBright for live imaging. The sample is tumor sample.

**Supplementary Movie 7:** The time-lapse imaging of patient derived explant cultures (PDEC) after 6 days of ex vivo culture. The movie demonstrates the motility of tumor infiltrating lymphocytes (TIL) in Matrigel cultures. The TILs are stained with CD45+ VioBright for live imaging. The sample is tumor adjacent normal tissue.

**Supplementary Movie 8:** The time-lapse imaging of patient derived explant cultures (PDEC) after 6 days of ex vivo culture. The movie demonstrates the motility of tumor infiltrating lymphocytes (TIL) in Matrigel cultures. The TILs are stained with CD45+ VioBright for live imaging. The sample is tumor sample.

**Supplementary Movie 9:** The time-lapse imaging of patient derived explant cultures (PDEC) after 6 days of ex vivo culture. The movie demonstrates the motility of tumor infiltrating lymphocytes (TIL) in Matrigel cultures. The TILs are stained with CD45+ VioBright for live imaging. The sample is tumor sample.
