# Supplementary material for: Soft matrix promotes immunosuppression in tumor-resident immune cells via COX-FGF2 signaling
Source: Nat Commun. 2025 May 27;16:4908. doi: 10.1038/s41467-025-60092-x (PMC12116891; doi:10.1038/s41467-025-60092-x)
Supplement: Supplementary file 4 — Reporting Summary [file 41467_2025_60092_MOESM4_ESM.pdf]

nature portfolio

Corresponding author(s): Juha Klefström, Pauliina Munne

Last updated by author(s): 27.01.2025

Reporting Summary

Nature Portfolio wishes to improve the reproducibility of the work that we publish. This form provides structure for consistency and transparency in reporting. For further information on Nature Portfolio policies, see our Editorial Policies and the Editorial Policy Checklist.

Statistics

For all statistical analyses, confirm that the following items are present in the figure legend, table legend, main text, or Methods section.

n/a	Confirmed	
☐	☒	The exact sample size (*n*) for each experimental group/condition, given as a discrete number and unit of measurement
☐	☒	A statement on whether measurements were taken from distinct samples or whether the same sample was measured repeatedly
☐	☒	The statistical test(s) used AND whether they are one- or two-sided<br>*Only common tests should be described solely by name; describe more complex techniques in the Methods section.*
☒	☐	A description of all covariates tested
☐	☒	A description of any assumptions or corrections, such as tests of normality and adjustment for multiple comparisons
☐	☒	A full description of the statistical parameters including central tendency (e.g. means) or other basic estimates (e.g. regression coefficient) AND variation (e.g. standard deviation) or associated estimates of uncertainty (e.g. confidence intervals)
☐	☒	For null hypothesis testing, the test statistic (e.g. *F*, *t*, *r*) with confidence intervals, effect sizes, degrees of freedom and *P* value noted<br>*Give P values as exact values whenever suitable.*
☒	☐	For Bayesian analysis, information on the choice of priors and Markov chain Monte Carlo settings
☐	☒	For hierarchical and complex designs, identification of the appropriate level for tests and full reporting of outcomes
☐	☒	Estimates of effect sizes (e.g. Cohen's *d*, Pearson's *r*), indicating how they were calculated

Our web collection on statistics for biologists contains articles on many of the points above.

Software and code

Policy information about availability of computer code

Data collection	No software was used.
Data analysis	The data was analyzed with R-studio v. RStudio 2023.06.1+524 "Mountain Hydrangea" and R version 4.3.1 (2023-06-16) were used for analysis and visualization. For mRNA seq analysis, DEseq2 and EdgeR were used for the analysis and for the spatial and single cell analysis, Seurat by Satija lab was the key software for the analysis.

For manuscripts utilizing custom algorithms or software that are central to the research but not yet described in published literature, software must be made available to editors and reviewers. We strongly encourage code deposition in a community repository (e.g. GitHub). See the Nature Portfolio guidelines for submitting code & software for further information.

Data

Policy information about availability of data

All manuscripts must include a data availability statement. This statement should provide the following information, where applicable:

- Accession codes, unique identifiers, or web links for publicly available datasets
- A description of any restrictions on data availability
- For clinical datasets or third party data, please ensure that the statement adheres to our policy

The scRNA seq data has been deposited to Zenodo and can be accessed via following link: https://doi.org/10.5281/zenodo.11118899. The bulk mRNA seq data is provided in the source data table.

Research involving human participants, their data, or biological material

Policy information about studies with human participants or human data. See also policy information about sex, gender (identity/presentation), and sexual orientation and race, ethnicity and racism.

Reporting on sex and gender	This study consists only female subjects as it researches female breast cancer.
Reporting on race, ethnicity, or other socially relevant groupings	This study does not report the race or ethnicity.
Population characteristics	The study consists mostly samples derived from finnish women.
Recruitment	Primary breast tumor tissue was obtained from elective breast cancer surgeries from Helsinki University Hospital. Patients participated in the study by signing an informed consent form following the Declaration of Helsinki principles.
Ethics oversight	The study was conducted under ethical permit (243/13/03/02/2013/ TMK02157 and HUS/2697/2019 approved by the Helsinki University Hospital Ethical Committee).

Note that full information on the approval of the study protocol must also be provided in the manuscript.

Field-specific reporting

Please select the one below that is the best fit for your research. If you are not sure, read the appropriate sections before making your selection.

☒ Life sciences ☐ Behavioural & social sciences ☐ Ecological, evolutionary & environmental sciences

For a reference copy of the document with all sections, see nature.com/documents/nr-reporting-summary-flat.pdf

Life sciences study design

All studies must disclose on these points even when the disclosure is negative.

Sample size	In the experiments, each individual patient was considered as a sample. No replicates from patient were done as tumor material obtained from each patient is often very small.
Data exclusions	One individual sample was excluded from cytokine analysis and from multiplex image analysis, as it did not contain any immune cells and thus there were no cytokines to analyze, or immune cells to stain in the multiplex.
Replication	Each experiment was replicated at least with three individual patient samples. However, as patient material is more heterogenous than cell lines, exact numbers may not be replicable in another patient tumor.
Randomization	We did not randomize samples to experiment groups.
Blinding	Patient samples were derived from pathology department without any prior diagnosis or details.

Reporting for specific materials, systems and methods

We require information from authors about some types of materials, experimental systems and methods used in many studies. Here, indicate whether each material, system or method listed is relevant to your study. If you are not sure if a list item applies to your research, read the appropriate section before selecting a response.

Materials & experimental systems

n/a	Involved in the study	
☐	☒	Antibodies
☒	☐	Eukaryotic cell lines
☒	☐	Palaeontology and archaeology
☒	☐	Animals and other organisms
☒	☐	Clinical data
☐	☒	Dual use research of concern
☒	☐	Plants

Methods

n/a	Involved in the study	
☒	☐	ChIP-seq
☐	☒	Flow cytometry
☒	☐	MRI-based neuroimaging

Antibodies

Antibodies used	Antibody list has been provided in the supplementary
Validation	Antibodies used in this study for immunohistochemistry were validated in the Juha Klefstom's research laboratory by using negative and positive controls, and serial dilutions of the antibodies. Western blot and immunofluorescent antibodies were typically validated using positive and negative controls.

Dual use research of concern

Policy information about dual use research of concern

Hazards

Could the accidental, deliberate or reckless misuse of agents or technologies generated in the work, or the application of information presented in the manuscript, pose a threat to:

No	Yes	
☒	☐	Public health
☒	☐	National security
☒	☐	Crops and/or livestock
☒	☐	Ecosystems
☒	☐	Any other significant area

Experiments of concern

Does the work involve any of these experiments of concern:

No	Yes	
☒	☐	Demonstrate how to render a vaccine ineffective
☒	☐	Confer resistance to therapeutically useful antibiotics or antiviral agents
☒	☐	Enhance the virulence of a pathogen or render a nonpathogen virulent
☒	☐	Increase transmissibility of a pathogen
☒	☐	Alter the host range of a pathogen
☒	☐	Enable evasion of diagnostic/detection modalities
☒	☐	Enable the weaponization of a biological agent or toxin
☒	☐	Any other potentially harmful combination of experiments and agents

Plants

Seed stocks	*Report on the source of all seed stocks or other plant material used. If applicable, state the seed stock centre and catalogue number. If plant specimens were collected from the field, describe the collection location, date and sampling procedures.*
Novel plant genotypes	*Describe the methods by which all novel plant genotypes were produced. This includes those generated by transgenic approaches, gene editing, chemical/radiation-based mutagenesis and hybridization. For transgenic lines, describe the transformation method, the number of independent lines analyzed and the generation upon which experiments were performed. For gene-edited lines, describe the editor used, the endogenous sequence targeted for editing, the targeting guide RNA sequence (if applicable) and how the editor was applied.*
Authentication	*Describe any authentication procedures for each seed stock used or novel genotype generated. Describe any experiments used to assess the effect of a mutation and, where applicable, how potential secondary effects (e.g. second site T-DNA insertions, mosiacism, off-target gene editing) were examined.*

Flow Cytometry

Plots

Confirm that:

☒ The axis labels state the marker and fluorochrome used (e.g. CD4-FITC).

☒ The axis scales are clearly visible. Include numbers along axes only for bottom left plot of group (a 'group' is an analysis of identical markers).

☒ All plots are contour plots with outliers or pseudocolor plots.

☒ A numerical value for number of cells or percentage (with statistics) is provided.

Methodology

Sample preparation	PBMCs and PDEC samples were grown in 3D matrices (GrowDex 0.3%, GrowDex 1.0%, PeptiGel, and Matrigel). NC matrices (GrowDex) were dissociated by adding 1:2 GrowDase (UPM Biomedicals) enzyme diluted in Mammocult and incubating samples at +37°C for 1h. PeptiGel was degraded with Cell recovery solution (Corning) at +4°C for 1h or with 10 mg/mL pronase in PBS for 10 min at +37°C. Matrigel was enzymatically digested by adding 400 µL of undiluted Cultrex Organoid Harvesting Solution (Nordic Biosite, 3700-100-01) at +4°C for 1h. After enzymatic dilution of the growth matrix, the cells were separated by using a filter cap tube (Falcon™) and centrifugating the cells at 400g for 5min at +4 °C.<br><br>Harvested cells were stained with the primary antibodies at +4°C for 1h. CountBright Absolute Counting Beads (Invitrogen, C36950) were used for cell number counting.
Instrument	BD FACS Aria II (BD Biosciences) or with NovoCyte Quanteon
Software	FlowJo version 10.8
Cell population abundance	The cell population abundance was highly dependent on the patient sample, as individual tumors contain different numbers of immune cells.
Gating strategy	The gating strategy is visible in the supplementary data table. The boundaries between positive and negative cell populations varied between each marker, but were selected based on zebra blots. Same boundaries were always used for each sample (controls and all the treatments).

☒ Tick this box to confirm that a figure exemplifying the gating strategy is provided in the Supplementary Information.
